# Critical Raw Materials Saving by Protective Coatings under Extreme Conditions: A Review of Last Trends in Alloys and Coatings for Aerospace Engine Applications

**DOI:** 10.3390/ma14071656

**Published:** 2021-03-28

**Authors:** Maria Luisa Grilli, Daniele Valerini, Anca Elena Slobozeanu, Bogdan O. Postolnyi, Sebastian Balos, Antonella Rizzo, Radu Robert Piticescu

**Affiliations:** 1 ENEA—Italian National Agency for New Technologies, Energy and Sustainable Economic Development, Casaccia Research Centre, Via Anguillarese 301, 00123 Rome, Italy; 2 ENEA—Italian National Agency for New Technologies, Energy and Sustainable Economic Development, Brindisi Research Centre, S.S. 7 Appia—km 706, 72100 Brindisi, Italy; daniele.valerini@enea.it (D.V.); antonella.rizzo@enea.it (A.R.); 3 National R&D Institute for Nonferrous and Rare Metals—IMNR, 102 Biruintei Blvd, Pantelimon, 077145 Ilfov, Romania; rpiticescu@imnr.ro; 4 IFIMUP—Institute of Physics for Advanced Materials, Nanotechnology and Photonics, Department of Physics and Astronomy, Faculty of Sciences, The University of Porto, 687 Rua do Campo Alegre, 4169-007 Porto, Portugal; b.postolnyi@gmail.com; 5 Department of Nanoelectronics and Surface Modification, Sumy State University, 2 Rymskogo-Korsakova St., 40007 Sumy, Ukraine; 6 Department of Production Engineering, Faculty of Technical Science, University of Novi Sad, Novi Sad, Trg Dositeja Obradovica 6, 21000 Novi Sad, Serbia; sebab@uns.ac.rs

**Keywords:** critical raw materials, protective coatings, thermal barrier coatings, aerospace engine blades, Ni-superalloys, Ti-based alloys

## Abstract

Several applications, where extreme conditions occur, require the use of alloys often containing many critical elements. Due to the ever increasing prices of critical raw materials (CRMs) linked to their high supply risk, and because of their fundamental and large utilization in high tech products and applications, it is extremely important to find viable solutions to save CRMs usage. Apart from increasing processes’ efficiency, substitution, and recycling, one of the alternatives to preserve an alloy and increase its operating lifetime, thus saving the CRMs needed for its manufacturing, is to protect it by a suitable coating or a surface treatment. This review presents the most recent trends in coatings for application in high temperature alloys for aerospace engines. CRMs’ current and future saving scenarios in the alloys and coatings for the aerospace engine are also discussed. The overarching aim of this paper is to raise awareness on the CRMs issue related to the alloys and coating for aerospace, suggesting some mitigation measures without having the ambition nor to give a complete overview of the topic nor a turnkey solution.

## 1. Introduction

The first debates about the supply risk of strategic materials date back to the late 1930s, but in the last ten years, a great concern has arisen about the supply security of metals and other strategic elements, especially from import-dependent industrialized countries such as the EU, Japan, and USA, whose high-tech products are strongly dependent on them. Since 2011, the EU released a list of strategic elements and materials, the so called “Critical Raw Materials (CRMs) list” [1], which is updated every three years to take into account the evolving scenarios of demand from industry (economic importance) and supply risk of the critical elements. “*Economic importance looks in detail at the allocation of raw materials to end-uses based on industrial applications. Supply risk looks at the country-level concentration of global production of primary raw materials and sourcing to the EU, the governance of supplier countries, including environmental aspects, the contribution of recycling (i.e., secondary raw materials), substitution, EU import reliance and trade restrictions in third countries*” [2].

**Figure 1:**
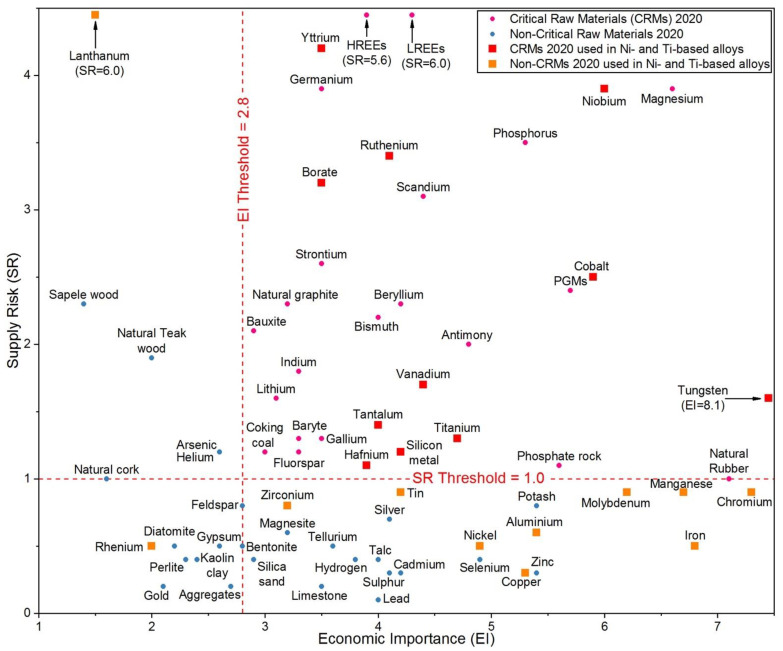
CRMs in 2020 for the EU (adapted from Ref. [3]). LREEs and HREEs stand for light and heavy rare earth elements (REEs), respectively.

Figure 1 reports the chart with all the materials considered in the last CRMs list released in 2020 [3], where the critical raw materials are marked in red (square) and pink (circle) colours.

It is noticeable how the deployment of renewable energy generation and e-mobility solutions has translated into raw materials demands, leading to “new entries” such as lithium, which was never included in the previous CRM lists. Moreover, many of the raw materials assessed in the 2020 list are also essential for the development of other strategic sectors such as defence and aerospace, robotics and digital technologies, and 3D manufacturing.

Jin et al. carried out a study on existing works about critical materials, analyzing methodologies for determining the critical raw materials and aiming to illustrate a criticality research area map as well as research gaps [4].

Ku and Hung recently introduced methods to identify at-risk, critical materials, and outlined general strategies to address challenges related to CRMs issues, analyzing some of the technical aspects associated with the production, processing, and recycling of such materials [5].

A systematic approach for materials selection in a critical raw material perspective, was recently proposed by Ferro and Bonollo, who developed a method allowing the selection of the alloy for the current application that minimizes its criticality associated with CRMs [6].

Saving CRMs can be achieved by their substitution, i.e., the reduction or the complete elimination of a particular CRM in the considered application. This can be obtained by replacing/reducing the critical element content in the alloy material by the use of non-critical alternative elements, or totally replacing the alloy itself and/or the whole fabrication process. Another way to save CRMs’ content avoiding their replacement, especially if the alloy is submitted to severe operating conditions, is by the application of surface coatings or surface treatments, therefore increasing the lifetime of the coated component and indirectly saving the CRMs consumption for the alloy’s manufacturing [7,8,9]. Additive manufacturing (AM) of metal alloys and the use of secondary raw materials (recovered through recycling technologies) are two other important approaches contributing to face CRMs shortage. However, recycling is out of the scope of the present manuscript, and additive manufacturing will be only briefly mentioned, being more extensively discussed in [10] by some of the authors of this manuscript. Our review will be manly focused on Ni- and Ti-based alloys used in aerospace turbines and, especially, on the use of coatings on these alloys.

**Figure 2:**
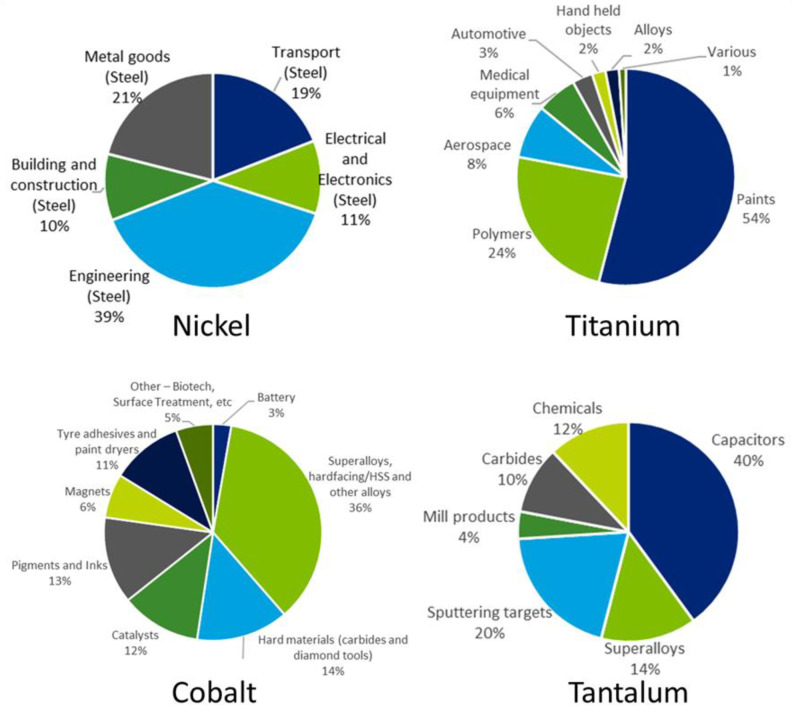
End uses of some materials contained in superalloys. Graphs taken from the EU reports [11,12].

Coatings are extremely useful to protect substrates materials when severe operating conditions occur, because they are able to enhance the performance of the coated materials to withstand high temperatures, corrosive environments, high wear rates, etc. Among the various extreme conditions, this paper is focused on high temperatures to which alloy materials for aerospace engine parts are submitted. Such alloys contain several critical and near critical elements such as Ni, Cr, Co, Mo, W, Ti, Ta, Nb, Hf, Si, C, Re, Ru, etc., and many coatings normally employed in aircraft engines for protection purposes contain CRMs as well. The end uses in EU for some of these elements as resulting from the 2020 EU reports [11,12] are shown in Figure 2.

Although nickel (Ni) is not included in the 2020 EU CRMs list, it is closely monitored by the EU Commission because its demand is expected to increase in the next years, mainly due to the growing market of batteries for electric vehicles. For this reason, it cannot be excluded that nickel will enter the CRMs list in the next years. In the EU market, nickel is mainly used for alloys, including alloyed steel and non-ferrous alloys for aerospace applications.

Titanium, cobalt, and tantalum are already included in the 2020 EU CRMs list. Titanium (Ti) entered the list only in 2020, and its economic importance is also justified by its import reliance close to 100% for the EU member states and by the increased range of applications as a result of its use in the transport sector (i.e., in titanium alloys used in turbine engines) during the 20th century. One of the most significant applications of Ti is in lightweight high-strength alloys for aeronautics, space, and defence, where its light weight results in better performance with lower fuel consumption. The use of cobalt (Co) in alloys, such as superalloys for gas turbine engines, is the major application for cobalt in the EU. Similarly, a great share of tantalum (Ta) consumption in the EU is dedicated to superalloys for the aerospace sector.

At the same time, other CRMs have significant importance for the use in alloys and superalloys for aeroengines. For example, even though the largest end use of tungsten (W) is in cemented carbides, it is often used in superalloys for engines and turbines. Around 18% of niobium (Nb) consumption in the EU is used for the production of alloys and superalloys, especially high strength low alloy (HSLA) steels for vehicles bodies and gas and oil pipelines, but also for turbines. Beryllium (Be) is used for several applications, like in defence, transportation, and energy. Thanks to its chemical, mechanical, and thermal properties, Be can be employed in low weight and high rigidity components like in the aerospace sector.

Another class of CRMs that finds usage in the aerospace sector is the one of REEs. Most of their use in the EU is aimed at applications in catalysts, glass, batteries, lighting devices, and magnets, but a significant amount is also used in metallurgy and ceramics. For example, gadolinium (Gd) is used in metallurgical applications for improving the mechanical characteristics of alloys, like workability and resistance to high temperature oxidation. Additionally, Yttrium (Y) is used as an additive in alloys to increase their strength, while its main use in the EU is in the production of refractory ceramics like yttria-stabilized zirconia (YSZ). Indeed, several REEs are even used in thermal barrier coatings, as shown in next paragraphs.

Chromium (Cr) and molybdenum (Mo) are quasi-critical materials, with a high economic importance for the EU and at the edge of criticality (see Figure 1), and they are both largely used in alloys and superalloys for the aerospace sector since they confer high-temperature and corrosion-resistance properties to the alloys.

Other elements, such as rhenium and ruthenium, are also worth mentioning in this framework, as they are expensive and rare materials that are used to improve the properties of alloys used in aerospace technologies. Rhenium (Re) is not included in the 2020 EU CRMs list, however it is one of the rarest elements in the Earth’s crust and more than 80% of Re consumption is dedicated to the aerospace sector, especially as dopant in superalloys to increase their resistance to creep and fatigue. Moreover, even if recycling can provide for a significant part of its demand, Re request is expected to increase in next years as a consequence of the increasing air traffic. Ruthenium (Ru) is a CRM belonging to the class of platinum group metals (PGMs). It is a rare element as well, and it is included in last generations of aerospace Ni superalloys to improve their thermal resistance, thus contributing to the high cost of these alloys.

It is then clear that, due to the high number of critical or quasi-critical materials contained in turbine engines and their economic importance for the EU markets, different solutions should be adopted to achieve effective CRMs saving. Apart from their complete substitution, which is often almost impossible or would require a drastic revolution in present industrial technologies and processes, the use of surface coatings or surface treatments represents a valuable strategy to this goal.

This paper deals with the analysis of the alloys and coatings for the aerospace engine, aiming at providing future perspectives about CRMs saving and CO_2_ reduction. Some results are also reported about an innovative thermal barrier coating (TBC) system obtained by doping zirconium oxide with mixed rare earth oxides (as yttrium oxide substitutes) occurring in the natural composition as extracted directly from monazite concentrate minerals through an efficient and green route [13]. This study, carried out by some of the authors of this manuscript in the frame of the EU ERA-MIN 2 project MONAMIX, New concepts for efficient extraction of mixed rare earths oxides from monazite concentrates and their potential use as dopant in high temperature coatings and sintered materials, is still in progress.

Due to vastness of the topics, this manuscript aims to provide an analysis of Ti- and Ni-based alloys and coatings for the aerospace engine from the CRM perspective, to raise awareness over CRMs issues, more than having the ambition to give an exhaustive overview on the topics and a turnkey solution. With respect to previously published works, mainly focused on a specific issue, a comprehensive study is reported here, encompassing several topics: CRMs, Ni-, and Ti-based alloys, coatings, surface treatments, and manufacturing processes.

## 2. Aircraft Engines and CRMs

### 2.1. Materials for Aircraft Engines

**Figure 3:**
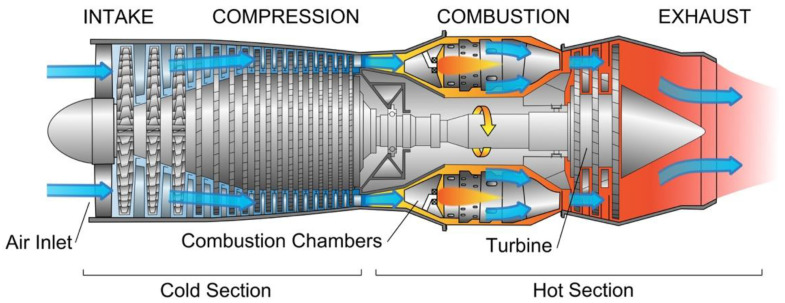
Schematic of a turbo jet engine [16].

The basic configuration of a turbojet engine is: (i) an air intake, (ii) a compressor or a fan, (iii) a combustor or a combustion chamber, (iv) a turbine, and (v) an exhaust nozzle (Figure 3). The engine operates by taking up air, compressing it into the smallest volume possible, mixing it with fuel, and burning it inside the combustion chamber. The ejected hot gases from the hot air–fuel mixture generate the force necessary to propel the aircraft. To increase the engine efficiency, attention to aerodynamic designs for compressor and turbines, search for improved cooling technology for turbine blades, and advances in alloys and coatings materials and manufacturing technologies are necessary to obtain engines with increased power, improved fuel efficiency, reduced CO_2_ and NO*_x_* emission, and reduced noise generation [14].

According to thermodynamics, the higher the combustion chamber temperature, the higher the performances, therefore as engines have become hotter, the materials for the combustion chamber have changed from heat-resistant steels to heat-resistant alloys containing nickel or cobalt [15].

In the compressor section, axial or radial according to the flow vein shape, where air taken into the engine by the fan is compressed, the materials generally used are Fe-, Ni- and Ti-based heat resistant alloys. The compressor can reach temperatures up to 1000 °C and the air pressure is raised up to 30 times. In the combustor section, where the compressed air is mixed with fuel and a high energy airflow is generated to propel the aircraft, extremely hot volumes of gases are produced, which are routed into the turbine engine and through the exhaust creating thrust. The temperature inside the combustor can reach 1500–1600 °C. The combusted gases from the combustion chamber go to the turbine section consisting of a high-pressure (HP) and a low-pressure (LP) turbine. The turbine is connected to the compressor through a shaft or spool which passes along the combustion chamber, and turbine’s blade extracts energy from the high-pressure gases. Therefore, blades and vanes of the high-pressure turbine section of aircraft jet engines are the most stressed components, submitted to harsh environments, high temperature and high load, i.e., high temperature gradients (700–1200 °C) and severe stresses (100–800 MPa). The high operation temperature can limit the performance of the turbine blades and therefore of the turbine engine itself, thereby making them more vulnerable to creep and corrosion failure, while vibrations in the turbine engine can result in fatigue failure [17]. For those high-temperature engine parts, Ni-based superalloys are nowadays the most widely used materials. However, advanced modern aeroengines can reach temperatures as high as 1600 °C in the hottest sections, thus exceeding the melting temperature of Ni-based superalloys (~1200–1300 °C). To overcome this limitation, many research studies are conducted to find possible alternative materials, such as silicide alloys, Co-based superalloys, Mo and Nb-based alloys, and refractory high entropy alloys (RHEAs) [18,19]. A very recent paper from Tsakiropoulos reviewed some of the last trends in the search for metallic materials alternative to Ni-based superalloys able to withstand the temperature of the hottest parts of future engines. In the “beyond the Ni-superalloys era”, transition metal TM and refractory metal RM aluminides and silicides with higher melting points (1800–1900 °C) were proposed [20]. Suitable alternative materials should fulfil several simultaneous requirements, such as the capability to withstand higher temperatures together with oxidation and corrosion resistance, fracture resistance, and light weight.

In order to withstand the high heat loads experienced in the combustor region of the engine during flight, actively cooled panels are employed. Herein, a fuel before being injected into the combustor serves as a coolant and is made to flow through the combustor heat exchanger panels such that the material and coolant temperatures are maintained below their critical limits. A few of the candidate materials considered for the active panels of the engine are Nb alloy Cb 752 and Ni alloy Inconel X-750. It was shown that Inconel X-750 is capable of sustaining high heat transfer coefficients with fuel/coolant heat gain well below fuel coking temperature with moderate weight to area ratio [21]. Vermaak and co-workers analyzed (in respect with Inconel X-750) the potential use of some other metallic materials for active cooled applications and reached the following conclusions: Cu-based alloy GRCop-84 requires a much bulkier structure to withstand the same thermomechanical environment, although its conductivity exceeds that for INCONEL X-750 by over an order of magnitude; the use of Ti-β21S alloy is limited due to its low softening temperature as well as the pressure induced stresses; Nb-based refractory alloy C-103 exhibits unexpected similarities with INCONEL X-750 [22].

Apart from cooling, whatever the base material for the hottest parts of the engine, an additional crucial step to keep the temperature of the components below the critical point is the realization of proper thermal barrier coatings. As a consequence, continuous improvements are sought through extensive research on the whole system made of superalloy/bond coat/thermal barrier [23].

Advanced aeroengine designs are recently oriented towards the reduction of specific fuel consumption (SFC) and increase of thrust-to-weight ratio, heat-resistance as well as light weight. Therefore, the nickel alloys used in the low-pressure part of the turbines are being substituted by titanium aluminide, which boasts superior properties [15]. TiAl-based alloys have several advantages such as low density, high stiffness, high yield strength, and good creep resistance under service temperatures up to 900 °C, however they suffer from oxidation corrosion at operating temperature exceeding 750 °C [24,25]. Additionally, in this case, the whole system Ti-based alloy/bond coat/thermal barrier must be optimized to guarantee Ti alloys to operate under harsh service conditions.

It is worth noting that the global market for the aircraft industry has a strong increasing trend. In 2019, Airbus forecasted an annual growth of 4.3%, requiring about 39,200 new passengers and dedicated freighter aircrafts over the next 20 years. Despite passenger travel activity decreasing sharply in 2020 due to the COVID-19 pandemic, a long-term recovery and a return to rapid growth in the aviation industry are expected [26]. Therefore, there is a growing need for new commercial airplanes with improved fuel efficiency and reduced pollution emissions [27]. Optimizing aircraft loads and thus decreasing fuel consumption and emissions per passenger, and improving aircrafts reliability will have therefore a very high impact on costs, environment, and safety. Because engines are the key components of aircrafts, improving the safety, reliability, and economy of engines is crucial. To this aim, machine learning is emerging as a promising tool for predicting the remaining useful life (RUL) of the aircraft engine, allowing previsions on operation and maintenance [28,29,30]. The role of machine learning in revolutionizing, redefining the modern scientific, technological, and industrial landscapes for aerospace engineering is reviewed in ref. [31].

Actually, the global AM market is worth 12 billion US dollars and is expected to reach about 30 billion US dollars in 2028. Machine learning technology is foreseen to make AM processes of metallic alloys for aerospace cheaper and faster [32]. AM techniques allow for reduced material waste and energy usage, easy prototyping, and optimization of components. The substrate metallic materials can be a wrought product, a forging, a casting, or a defective/damaged part [33]. For the aircraft industry, AM may be applied for the manufacture of components, the repair of components, and the manufacture and repair of tooling. Regarding blades and vanes, the potentialities of AM seems higher in repairing than on manufacturing of components, due to the little reduction in buy-to-fly ratio it may provide [33]. In October 2020, Satair, an Airbus subsidiary, delivered the A320ceo wingtip fences manufactured with the use of additive manufacturing, which was claimed as “*the first certified metal printed flying spare part*” [34].

However, several drawbacks should be solved before commercialization and further work is still required for AM process optimization. In fact, because AM can be described as a multilayer/repeated welding process, the manufactured component is susceptible to the formation of weld-cracking defects [35]. Moreover, the problems of high residual stresses due to rapid cooling rates associated with AM processes still must be solved.

**Figure 4:**
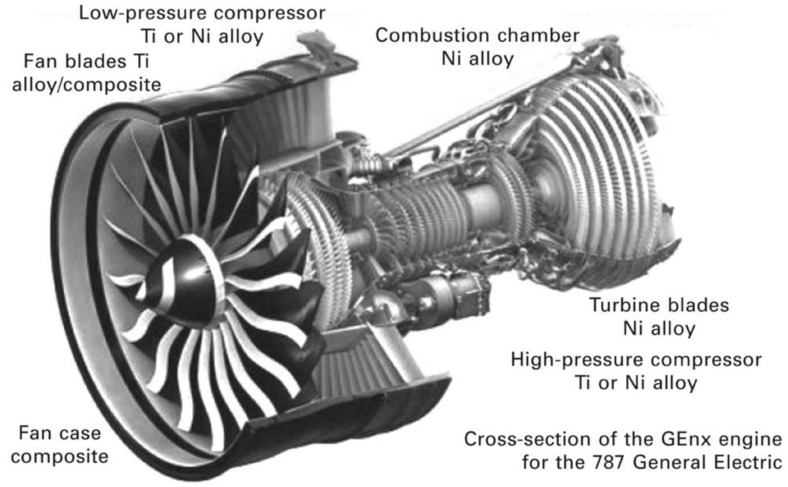
Distribution of Ni and Ti-based alloys in the aircraft engine. Reprinted from [36] with permission from Elsevier.

The next section will briefly describe the Ni-based superalloys and Ti-based alloys mostly used in compression and combustion chambers of jet engines (Figure 4), while Section 3 will review the coatings used for protection of these alloys.

### 2.2. Ni-Based Superalloys

**Figure 5:**
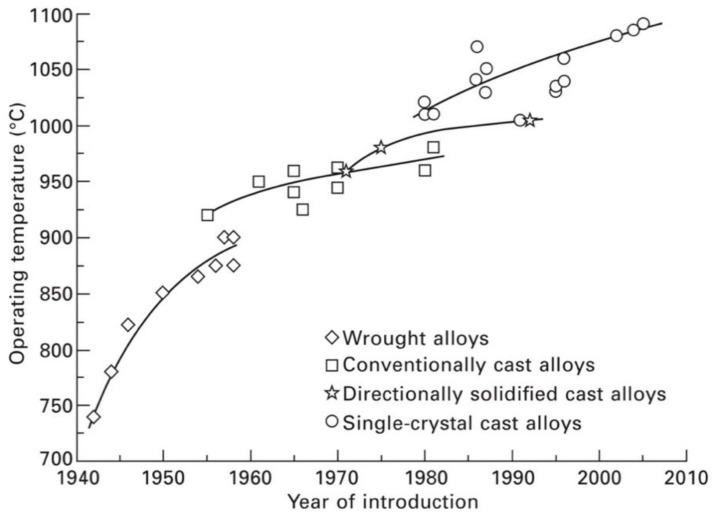
Temperature limit of superalloys for the aircraft turbine. Reprinted from [36] with permission from Elsevier.

Ni superalloys constitute about 50% of the total mass of advanced aircraft engine [37] and they represent the most used materials in turbine engines, thanks to their peculiar characteristics, such as great resistance against oxidation, corrosion, creep, and stress rupture at high temperature, providing them high strength and strong fatigue resistance under operating conditions. Compared to other alloys like aluminum and titanium alloys, used in lower-temperature zones and creeping at relatively low temperatures (e.g., above 150–350 °C), nickel superalloys can resist creep at several hundred degrees higher temperature [36]. Due to the complex composition and microstructure of Ni-based superalloys, the resulting properties of the final component (turbine blade or other elements in the aeroengine) are strongly dependent on the fabrication process employed. Based on the fabrication method, Ni superalloys are generally classified into wrought and cast superalloys, but the casting techniques strongly evolved from the early sixties onwards (Figure 5), allowing the achievement of better performing alloys. Briefly summarizing a typical process for the fabrication of a superalloy piece, the initial step is melting the base raw materials, like in the vacuum induction melting (VIM) stage. Then, the resulting alloy can be directly passed to the casting and wrought stage, or subjected to some additional remelting steps, for example, by means of arc remelting, in order to improve the grain size and reduce particle segregation before the successive casting stages. Through the use of conventional casting processes, the resulting components present a polycrystalline structure that, however, is limited to low temperature resistance (Figure 5). The subsequent introduction of advanced casting methods, like the directional solidification (DS) and single crystal (SX) production techniques, has played a fundamental role in increasing the strength of HP turbine blade alloys against creep and fatigue during service.

As an alternative to the casting process of the melted alloy, powder metallurgy is also employed for the fabrication of single crystal turbine blades. In this technique, superalloy powders obtained from the initial melting stage are then further processed and consolidated by hot isostatic pressing (HIP) [38,39]. This method is reported to allow alloying more elements with uniform composition, grain size, and phase composition, and with reduced segregation and porosity. However other side effects can be obtained, like contamination or prior particle boundaries (PPB). As further steps before the completion of the final component, the casted or HIP alloy must be subjected to heat and aging treatments to improve its mechanical properties.

The trend toward production of metallic materials in powder form has also fostered the development of AM approaches for the synthesis of engine components, enabling the fabrication of geometrically complex parts in near net shape form with rapid transfer of 3D designs to final components. Despite the microstructure of Ni-superalloys parts manufactured by AM being different from conventional cast and wrought ones, due to a dendrite-like structure (seen also in cast alloys) which affects the isotropy of the alloy’s properties, post-processing treatments, for example HIP and aging heat treatments, may be applied to heal the defects caused by the AM process.

**Figure 6:**
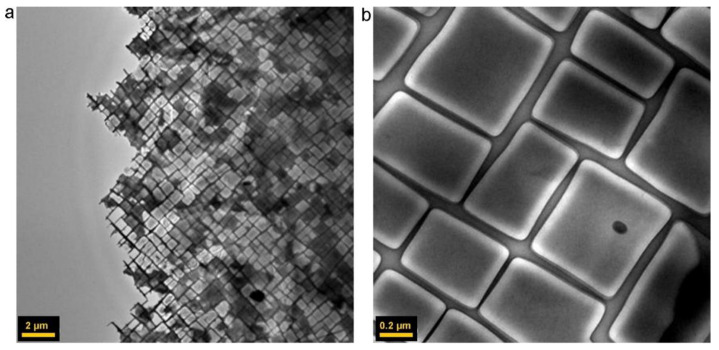
TEM images showing the typical structure of a Ni-based superalloy at low (**a**) and high (**b**) magnification, with the γ phase constituting the matrix channels and the γ’ phase constituting the cuboids. Reprinted from [43] with permission from Elsevier. Copyright 2012 ©Elsevier.

**Figure 7:**
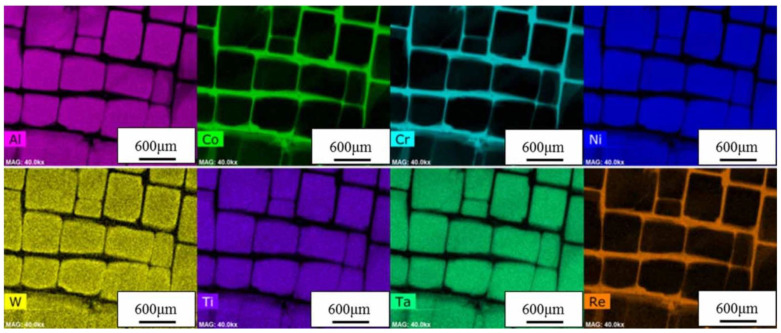
STEM-EDX (scanning transmission electron microscopy-Energy Dispersive X-Ray Analysis) maps showing the elemental distribution in the γ phase channels and in the γ’ phase cubes of a Ni-based superalloy. Reprinted from [44]. Copyright ©Institute of Materials, Minerals and Mining. Reprinted by permission of Informa UK Limited, trading as Taylor & Francis Group, www.tandfonline.com on behalf of Institute of Materials, Minerals and Mining.

Table 1 reports the most used Ni-based superalloys for engine applications [40,41,42]. The excellent mechanical properties of Ni-based superalloys are related to their microstructure. They have a characteristic two-phase microstructure formed by a γ matrix phase with a face-centred cubic (FCC) structure and a cuboidal γ′ precipitate that exhibits an FCC-based L12-ordered structure (Figure 6) [43]. Ni-based superalloys may contain up to 15 elements and most of them are CRMs. As shown in Figure 7, several elements like W, Co, Re, Cr, Mo, Fe, Y, etc., “many of which CRMs”, can act as alloying elements that stabilize the γ phase and promote the solution strengthening [44,45].

On the contrary, other elements can promote the formation of the γ’ phase, which is responsible for precipitation strengthening. The main solutes in Ni-based superalloys are Al, Ti, Ta, etc., with a total concentration that is generally less than 10 at %, required to form the characteristic γ’ phase, an intermetallic compound according to the formula Ni_3_(Al,Ti,Ta) [7,40]. The γ’ phase is largely responsible for the elevated-temperature strength of the material and its high resistance to creep deformation. Additional strengthening at low temperatures can be achieved by the γ” phase with the composition Ni_3_Nb or Ni_3_V in case of additions of Nb (Inconel) or V, by solid solution strengthening of γ phase, and by oxide dispersion strengthening (ODS). Molybdenum is added to the γ’ phase of Ni-based alloys as a strengthener at both room and elevated temperatures [46], while addition of niobium, titanium, and carbide formers is used to stabilize the alloys against the effect of chromium-carbide sensitization. The addition of a small quantity of chromium improves the oxidation and corrosion resistance at high temperature thanks to the formation of a Cr_2_O_3_ scale [17]. Indeed, the formation of chemically stable oxides, like Cr_2_O_3_ and Al_2_O_3_ scales, on the Ni-based alloy surface is known as a factor protecting the underlying alloy component from further high-temperature oxidation, thus aiding its high temperature resistance [44,47].

The single-crystal blades have superior creep resistance due to the fact that they are free from γ/γ′ grain boundaries. These boundaries are, in fact, an easy diffusion path which reduces the resistance of the material to creep deformation. Despite the directionally solidified columnar grain structure possessing many parallel γ grains, the boundaries are mostly parallel to the major stress axis and therefore the performance of such blades is not as good as the single-crystal blades [48].

The single-crystal superalloys are often classified into first, second, and third generation alloys according to the percentage of Re contained. The second and third generations contain about 3 and 6 wt.% of rhenium, respectively. Rhenium is a very expensive addition but leads to an improvement in the creep strength and fatigue resistance. Creep strength of the second and third generation alloys can be improved, respectively, by approximately 30 and 60 °C compared to the first generation alloys [49,50]. However, after a long exposure at high temperatures, creep performances are degraded by the formation of topologically closed-packed (TCP) phases. In the fourth generation Ni superalloys, a mass fraction of 2–3% Ru (a platinum group metal element, critical as well) is added to hinder the precipitation of TCP phases [51,52] and improve the high-temperature microstructure stability. In fourth generation superalloys, the creep strength increases, so that these alloys have capabilities to stand higher temperatures with respect to the previous generation superalloys. In fifth generation superalloys, the increase of Ru content (up to 5–6%) together with the accurate control of γ and γ’ phases, allows further increase of creep strength [50]. These benefits are unfortunately accompanied by an increase of density and cost of the alloys, as Re and Ru are expensive rare elements present in trace quantities in the Earth’s crust. Efforts are devoted to find viable substitutes for rare elements in the alloy composition without compromising the alloy’s original properties [53,54]. For instance, General Electric (GE) developed a low-Re superalloy René N515 with considerably reduced rhenium content (1.2 wt.% Re) and with comparable mechanical properties to the second generation René N5 (3 wt.% Re). In fact, GE recently assessed the critical materials used in its manufacturing and commercial operations, and responded to shortages of rhenium by minimizing the amount of critical metals in superalloys, giving an example of successful strategies for combating raw material criticality [5,26,55].

Commercialization of fourth and fifth generation Ni-based superalloys is prevented by their reduced resistance against oxidation due to the increased content of refractory metals, such as Ru, Mo, and Re. These refractory-based oxide species have relatively higher vapor pressures that can disrupt the continuity of protective Al_2_O_3_ formed on the surface during thermal exposure [51]. Quite recently, a sixth generation single crystal Ni-based superalloy, the TMS-238, has been developed for applications in gas turbine blades [51]. This alloy has both high-temperature creep strength and improved oxidation resistance and is able to achieve 1000 h of creep life at 137 MPa and 1120 °C [56]. TMS-238 alloy has 60% of its raw material costs determined by rhenium and 30% by ruthenium, for a total cost of about 2400 USD per liter [26]. Up to now, sixth generation superalloys of the TMS series have been developed by finely tuning the alloying elements in order to enhance the oxidation-resistance at high temperature and improve the creep strength. To this aim, computation-aided design methods have been developed [57,58]. The addition of a small amount of Si element (<0.5 wt.%) could help the formation of compact oxide scales, while excessive Si amount is detrimental to the mechanical property since it could accelerate the precipitation of TCP phases. The addition of Mo element (~0.6 wt.%) could improve the thermomechanical fatigue property of alloys. Unfortunately, all these improvements are at the expense of the creep rupture lifetime of the superalloy [57].

**Figure 8:**
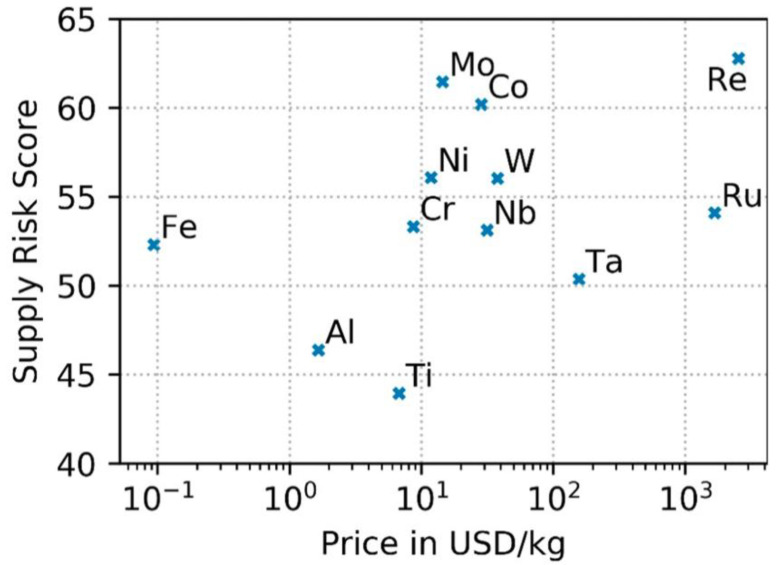
Supply risk score of elements in Ni-based alloys and the raw material price in a semi-logarithmic plot. Reprinted from [26] under an open access CC BY 4.0 license.

Recently, Helbig et al. evaluated the supply risks associated with elements contained in average Ni-based superalloys (Figure 8). On the basis of twelve indicators in four supply risk categories, rhenium, molybdenum, and cobalt were found to have the highest supply risk scores, while titanium and aluminum the lowest [26].

With the increasing temperature reached in modern advanced aeroengines, materials with still higher temperature resistance are requested. As a consequence, apart from the continuous search in the improvement of Ni-based superalloys to increase their high temperature performance while preserving their lifetime and mitigate the risk of CRMs shortage, intensive research is devoted also to the search of alternative materials. For example, ceramic matrix composites (CMCs) are studied thanks to their high-strength, low-weight, and high-temperature capability. In 2010, GE reported for the first time the development of CMCs-based turbine blades. The introduction of CMCs enables a fuel burn reduction up to two percent, difficult to reach in few other technologies in today’s pipeline. Moreover, the material density of CMCs is one-third that of today’s Ni-based alloys, enabling over 50 percent reduction in the turbine component weight [59]. CMCs were introduced commercially in high-pressure turbine shrouds, used in Airbus and Boeing and aircrafts.

Another promising research field is high entropy alloys (HEAs), which may provide a higher strength and fracture toughness and a higher heat resistance.

HEAs represent the class of alloys with the highest fracture toughness among the alloys developed so far, still keeping an acceptable yield strength, similar to that of Ni-based superalloys. When looking to the temperature dependence of the HEAs mechanical properties as classified in [60], only some HEAs can be suitable for the highest temperatures reached in turbine blades. For example, HEA-1 alloys, based on 3d-transition metals, do not provide any significant advance in terms of high temperature yield strength with respect to Ni-based superalloys. HEA-2 type alloys, based on transition metals with larger atomic-radius elements, present an increased yield strength only in a moderate temperature range (up to about 1000 °C), thus being unsuitable for the hottest regions of aircraft engines. On the contrary, the highest heat resistance is found in the case of HEA-3 type, characterized by addition of hafnium and highly refractory metals (Ta, W, Nb, Mo, etc.), allowing an increased yield strength at the highest temperatures. As a consequence, high entropy alloys are excellent materials candidates for compressors, combustion chambers, exhaust nozzle and gas turbine case applications within the gas turbine engine [61].

It is very likely, however, that HEAs may contain many critical raw elements. Appropriate screening methods could allow the selection of HEAs with a reduced CRM content [8,62,63] and AM optimized processes may provide further CRMs saving.

### 2.3. Ti-Based Alloys

Today, the aerospace industry is one of the world’s largest purchasers of titanium and titanium alloys. In particular, alloys based on TiAl have been investigated since the 1950s as advanced high temperature structural materials in the aerospace and marine industries, because of their light weight (~3.9–4.2 g/cm^3^, approximately half of that of nickel alloys), high specific yield strength and stiffness, and good creep resistance at elevated temperatures. It is worth reminding that, despite titanium being a vital ingredient in a plethora of other applications apart from aeronautics, space, and defence, it entered the CRM list only in 2020. The efforts in deploying Ti-based alloys for commercial aviation is motivated by the growing demand for new commercial aircrafts capable of satisfying the requirement of Aviation programs ACARE 2020 (Advisory Council for Aviation Research and Innovation in the EU) and Flightpath 2050 requiring for aircrafts a reduction of fuel consumption as well as CO_2_ and NO*_x_* emissions in the next 30 years. It is worth noting that air transport currently accounts for around 2% of the 36 billion tonnes of CO_2_ generated annually by human activities and, starting from 2020, the Flightpath 2050 targets to a 75% cut in CO_2_, a 90% reduction in NO*_x_*, and a 65% reduction in noise [64].

Among the various Ti-containing alloys, those based on titanium aluminide (TiAl-based alloys) exhibit better oxidation resistance than other Ti alloys, providing comparable strength as Ni-based alloys in the range of temperatures of 600–800 °C. Despite the enormous potential for structural application, a major drawback of many TiAl alloys is the low room temperature ductility and the poor castability, which make fabrication costs high and mass production difficult [65]. The poor room temperature ductility of the TiAl compound is due to its phase composition: α_2_-Ti_3_Al and γ-TiAl, not containing a sufficient number of primary dislocation slip systems for large-scale plastic deformation. Moreover, during high temperature exposure, the oxygen uptake from the α_2_ phase makes this alloy even more brittle due to further blocking of dislocation movement by oxygen interstitials, causing strain at fracture to drop from around 1.5% to less than 0.5% [66]. There are three generic types of microstructures: a fully or near α_2_/γ lamellar microstructure, an equiaxed γ grain microstructure, and a duplex microstructure that consists of α_2_/γ lamellar colonies and single-phase γ grains [65].

**Figure 9:**
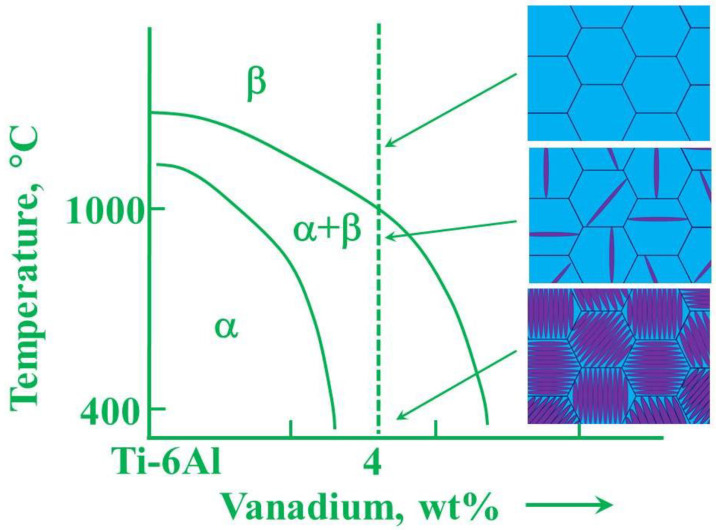
Idealized microstructures of TiAl alloys that arise due to forging in the β phase field and during subsequent cooling. Reprinted with permission from M. J. Whiting [68].

Ti alloys can be divided into three main classes on the basis of the phases that are present at room temperature (Figure 9), namely the hexagonal close-packed (hcp) α-phase, the body-centered cubic (bcc) β-phase, and the (α + β) dual-phase, together with other intermediate and metastable phases. The type of phase depends on the content of the various alloy elements and, ultimately, on the molybdenum equivalent (Mo_eq_) value.

In α-type alloys, the alloying elements guarantee an effective increase in the mechanical characteristics of the compact hexagonal crystal structure with a strengthening mechanism for a solid solution. Among the α-stabilizers elements, apart from aluminum and oxygen, there are nitrogen and carbon and neutral elements such as tin and zirconium. The allotropic transformation temperature in pure Ti, known as β-transus temperature, is at about 882 °C. In Ti alloys, the β-transus temperature changes depending on the alloying elements. The β-phase is stable above the β-transus temperature, while metastable and mixed phases are formed below the β-transus temperature, between the stable α and β phases. Alloying elements, that are considered α-stabilizers, tend to increase the β-transus temperature, stabilizing the α phase at higher temperatures, while the addition of β-stabilizers, such as Nb, Ta, Mo, V, Cu, Co, Cr, Ni tends to favor the β-phase formation at temperatures lower than 882 °C [67]. In Ti alloys, the β/α interfaces block the dislocation motion, therefore, the mechanical strength of any (α + β) microstructure depends on size, morphology, and distribution of α phase. The alloy elements in the β-phase are in the range 4–6%. Because the α-phase has a hexagonal crystal structure, it is stiffer and has less slip systems than the high-symmetry cubic crystal structure possessing multiple slip permitted by β-phase (bcc), thus, it has a limited conformability. However, the α-and near α phases possess higher creep and oxidation resistance with respect to the β-phase, therefore, α-phase alloys, such as Ti-3Al-2.5V, cp-Ti, Ti-5-2.5, Ti-8-1-1, Ti-6-2-4-2S, IMI829 are commonly used to make compressor disks and blades of aeronautic engines.

In the aerospace sector, however, Ti-6Al-4V (Ti-64), the first successfully developed Ti (α + β) alloy for aerospace purposes, dominates the scene. This alloy is about half of all titanium alloys industrially produced. In these alloys, aluminum is added as α-phase stabilizer and hardener due to its solution strengthening effect, while vanadium stabilizes the ductile β-phase, providing hot workability of the alloy [69]. Ti-6Al-4V alloy was developed for aircraft structural applications in 1950 and is one of the most extensively employed Ti-based alloys, especially in aerospace due to its superplasticity, allowing its production as sheets and, to a lesser extent, plates using standard production methods [70]. Beta titanium alloys have been available since the 1950s as well, but their significant usage did not occur until the mid-1980s on the B-1B bomber (in which Ti-15V-3Cr-3Al-3Sn was used) and in Boeing series undercarriages aircrafts, where Ti-10V-2Fe-3Al (Ti-1023) was extensively used, leading to a significant reduction in aircraft weight. Beta alloys with their unique characteristics such as excellent hardenability, heat treatability to high strength levels, and a high degree of sheet formability, are becoming increasingly important for the aerospace sector, for high-stressed aircraft components, e.g., landing gear and springs [71]. Over the years, an evolutionary trend in alloy design is also observed from the (α + β) alloys to the elevated temperature near-α alloys, which contain a low amount of β stabilizer. Near-α (or super α) Ti alloys are high-temperature Ti alloys with Ti-Al-Zr-Sn-Mo-Nb-Si alloying system whose β stabilizers (Mo, Nb and Si) are not more than 2%, being the Si element up to 0.5% [72,73]. These stabilizers introduce a small β phase and silicide precipitates in the microstructure, allowing good thermo-mechanical properties and creep resistances suitable for application in aircraft engine [74].

The different compositions and microstructures obtained then affect the resulting characteristics of the alloys. For example, increased yield strength without a corresponding strong decrease of ductility has been obtained in Ti-10V-2Fe-3Al alloy by Ellyson et al. through a phase strengthening deriving from nanoscale precipitates produced by aging and transformation induced plasticity (TRIP) [75]. A recent paper describes some relatively recent developments on the production and use of Ti and its alloys in aerospace components [76].

Since 1950, efforts have been devoted towards continuous increase of high temperature performances, leading to the application of several Ti-based alloys such as: Ti-6Al-2Sn-4Zr-2Mo-0.1Si (Ti-6242S) in 1960s and Ti-5.8Al-4Sn-3.5Zr-0.7Nb-0.5Mo-0.35Si (IMI834) in 1984. Afterward, Ti-6.2Al-2Sn-3.6Zr-0.7Mo-0.1Y-5.0W-0.5Si (BT36) and Ti–5.8Al-4.8Sn-2.0Zr-1.0Mo-0.35Si-0.85Nd (Ti60) with service temperature of about 600 °C were developed.

In 2006, General Electric announced the use of Ti-48Al-2Nb-2Cr (Ti-48-2-2), for making low pressure turbine (LPT) blades for their GEnx™ engine, being the first time that a TiAl alloy was employed for rotating parts of a commercial aircraft engine. This alloy, was successful used in high-thrust GEnx jet engines for LPT blade materials in Boeing 747-8 and 787 Dreamliner, entered into commercial service in 2012 in substitution of Ni-based superalloys in the temperature range of 650–750 °C with the benefit of a weight reduction of about 50% [77]. GEnx engine allows up to 15% improved fuel efficiency, 15% less CO_2_ compared to GE’s CF6 engine, and about 30% of noise reduction [78].

Casting offers the most cost-effective and well-known route for production of complex TiAl shapes, including turbine blades, being casting an already well-established route for Ti alloys. However, for casting TiAl alloys, low temperature is used because of the high reactivity of molten TiAl with usually employed ceramic crucibles. Additionally, the great affinity to oxygen of TiAl-based alloys prepared by conventional casting and forging methods makes difficult and expensive their recycling process. Novel manufacturing pathways via isothermal or nonconventional forging and powder metallurgical routes are currently under intensive research [79,80,81].

Recent advances on titanium aluminide have been done by Chen et al. who used a seedless growth approach to manufacture single crystals of a TiAl alloy with exceptional combination of ductility and strength, as well as resistance to creep up to 900 °C [82]. Addition of high amounts of silicon to TiAl alloy leads to a strong improvement of the oxidation behavior [83,84].

A review of opportunities and issues in the application of titanium alloys for aerospace components is found in ref. [85].

Improvements in the properties of Ti- and TiAl-based alloys can be obtained by subsequent post-processing methods. For example, in a recent paper by A.S. Chauhan et al. [86], laser surface heat treatment was tuned on a VT3-1 (α+β) titanium alloy to favor the formation of a microstructure surface gradient with a β-reach phase with high hardness. In another recent work by A. Bhardwaj et al. [87], high-temperature constrained groove pressing (CGP) was applied on Ti-6Al-4V alloy, providing an increase in a yield strength, tensile strength and microhardness.

Additive manufacturing of Ti-alloys is also a very active research field with high potential for application in aerospace engines. In 2015, GE Aviation used laser-printed fuel nozzles for LEAP (Leading Edge Aviation Propulsion) engines with a 25% reduction of weight and reduced the number of components from 20 to 1. GE Aviation also developed Ti-based blades, heat exchangers, and other parts for GE9X engine developed for Boeing’s new 777X. The most widely investigated AM TiAl-based alloy is Ti-6Al-4V. However, additive manufactured components often do not exhibit the same quality of components made by conventional methods, due to occurrence of internal defects (e.g., porosity) and formation of a complex microstructure, thus affecting the resulting fatigue and other mechanical properties. For example, Zhang et al. [88] recently observed the presence of α lamellae with six variants, together with multiple sub-variants, whose relative content influenced the mechanical anisotropy in a Ti-6Al-4V alloy manufactured via selective electron beam melting (SEBM). Post-processing applied on additive manufactured Ti-6Al-4V components show enormous potential in improvement of the alloy’s quality, e.g., by HIP process which leads to a homogenous microstructure and can reduce the porosity of titanium alloy parts produced by selective laser melting (SLM) [89]. In addition to internal defects and inhomogeneous microstructure, alloys fabricated by AM processes like laser powder bed fusion (L-PBF) and electron beam powder bed fusion (EB-PBF) can present a rough surface, where surface features can act as stress concentration factors that are responsible for strong reduction of fatigue strength with respect to conventionally manufactured materials [90]. Then, this aspect often requires additional surface post-processing stages on additive manufactured components, like surface machining, electropolishing, chemical polishing, and shot peening. Different surface post-processing methods on L-PBF and EB-PBF Ti-6Al-4V alloys have been analyzed in a recent work by Kahlin et al. [90] in order to reduce surface roughness and control the residual stresses in the material, so to increase the fatigue life of the alloy. A specific paragraph dedicated to laser shot peening (LSP) on Ti-based alloys is reported later.

## 3. Coatings for Ni- and Ti-Based Alloys for Aerospace Engine

### 3.1. Thermal Barrier Coatings for Ni-Based Superalloys

**Figure 10:**
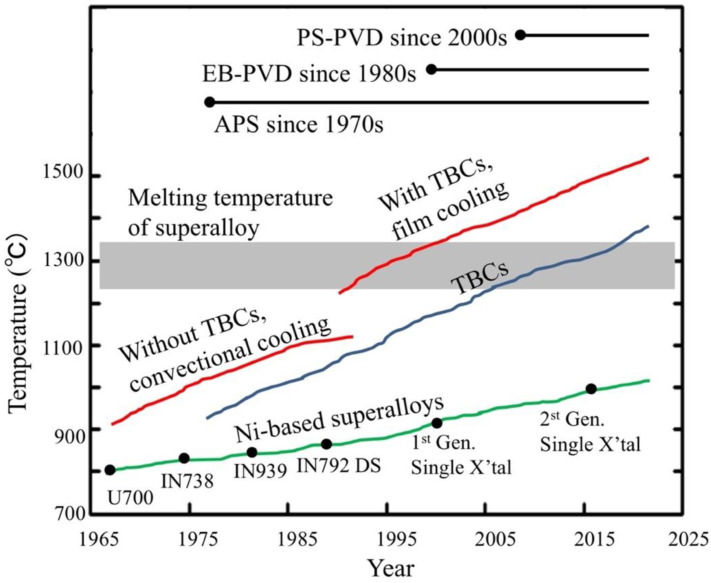
Thermal barrier coating progress for engine turbine. Acronyms stand for: PS-PVD = plasma spray-physical vapor deposition; EB-PVD = electron beam-physical vapor deposition; APS = atomic plasma spray. Reprinted from [92] under an open access CC BY 4.0 license.

As previously mentioned, aerospace components of the hot sections of the engine must be protected by a reliable thermal barrier coating which allows them to withstand their severe working conditions. Thanks to the use of cooling systems (mentioned above) and of TBCs, increase of the engine operating temperature and thus of fuel efficiency conversion, has been possible at temperature higher than the alloys’ melting point (Figure 10). It is worth noting, that a service temperature increase of 200 °C may enhance the turbine efficiency of 5–6%, leading to an important reduction of CO_2_ and NO*_x_* emission in the atmosphere [91].

Thermal barrier coatings may be effective in prolonging the alloys’ lifetime, enabling their safe operation in harsh conditions such as extreme high/low temperatures, high thermal shocks, high pressures and mechanical stresses, radiation or corrosion, therefore helping to save CRMs consumption. The coatings act as diffusion barriers to slow down the reaction between the substrate material and the aggressive environment [93]. However, during service operation, together with hot corrosion and oxidation, problems related to coating inter-diffusion on the substrate alloy and Al empowerment of the substrate alloy may from one side modify the mechanical properties of substrate alloy and from the other side reduce the oxidation resistance of the coating [94].

In gas turbines, actual components are not submitted to pure creep loading. In fact, due to the start-up and shut-down operations, transient temperature gradients occur, giving rise to combined thermal and mechanical loading [95], and therefore to thermal mechanical fatigue (TMF) and low cycle fatigue (LCF) [95,96]. High-temperature LCF in nickel-based alloys under air results in the formation of a complex oxide layer and a reaction zone depleted of strengthening precipitates in the adjacent substrate as in creep [97]. Under thermal transients, oxidation embrittlement can occur, therefore high-temperature LCF tests and TMF life prediction of alloys (coated and uncoated) for high-pressure turbine blades are mandatory [98].

**Figure 11:**
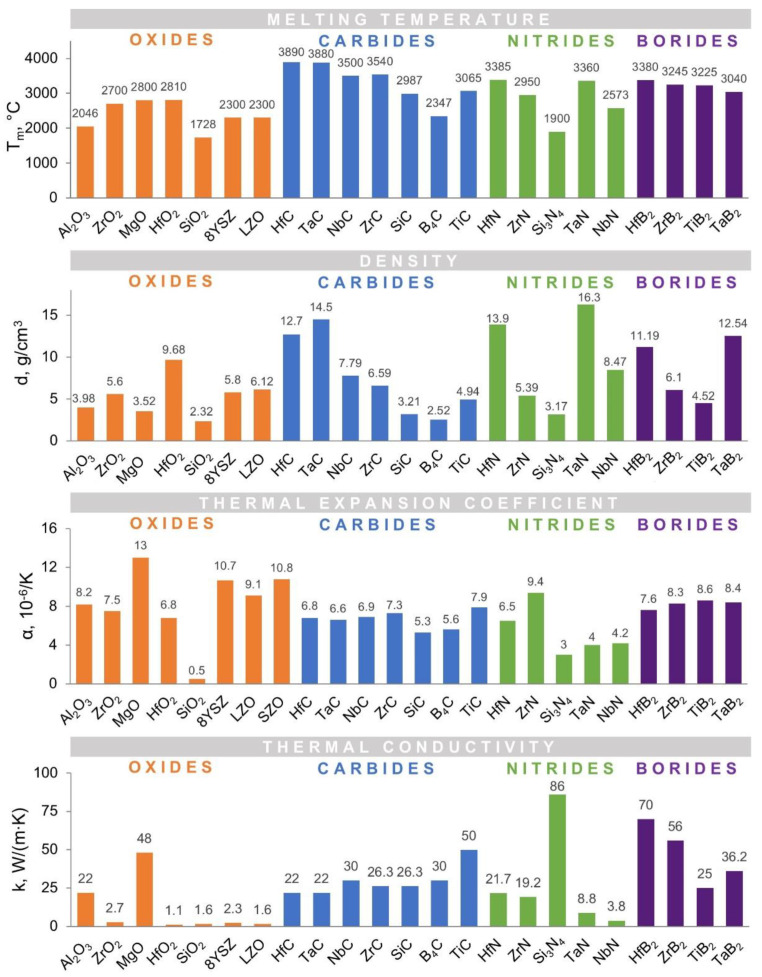
Properties of oxides, carbides, nitrides and borides required for TBC (Tm—melting temperature; d—density, α—thermal expansion coefficient, k—thermal conductivity). Figure is based on data taken from [104].

The number of materials that can be used as TBCs for extreme environments is restricted due to some critical requirements: (i) good thermal insulation, i.e., low thermal conductivity and low transparency to thermal radiation, (ii) high melting point, (iii) chemical inertness, (iv) no phase transformation between room temperature and operation temperature, (v) high thermal expansion to reduce thermal stress, (vi) good match with underlying metallic substrate and thermally grown oxide, (vii) hot corrosion resistance and (viii) erosion resistance. Ultra-high temperature ceramics (UHTC) displaying a unique set of properties, including extremely high melting temperatures (close to 3000 °C), such as carbides, nitrides, borides, and special oxides of the transition metals are promising candidates for thermal protection systems (TPS) [99,100]. For all the mentioned materials for application as TBCs, the thermal conductivity is a critical parameter that serves the main purpose, namely to reduce the surface temperature of the coated element [101]. Figure 11 shows some of the main properties of UHTC with potential applications in TBCs.

**Figure 12:**
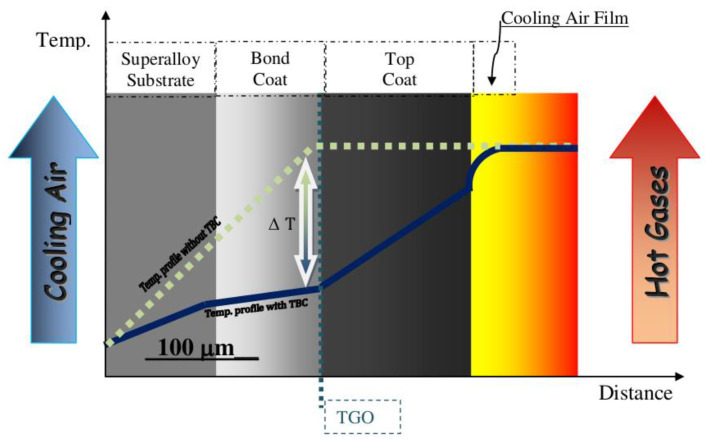
Scheme of a typical thermal barrier coating.

The simplest thermal barrier coating system used to protect Ni-superalloys is shown in Figure 12. It consists of two key layers: an oxidation resistant bond coat such as diffusion aluminide (based on intermetallic compound β-NiAl grown by vapor phase aluminizing (VPA) or chemical vapor deposition (CVD)) or overlay MCrAlY bond coating (grown by EB-PVD, air, vacuum, and low-pressure plasma spraying or high velocity oxy-fuel) and a ceramic top coat, being actually YSZ the preferred material [102]. The bond coat (BC) is deposited between the metallic substrate and the top coat (TC) to protect the underlying superalloys from oxidation and high temperature corrosion and to assist the coupling of the ceramic top coat and the metallic substrate, balancing the thermal mismatch between the top coat and the substrate. A thermally grown oxide (TGO) scale, predominantly alumina, is formed during service at elevated temperature as the reaction product between the top coat and bond coat (oxidation of BC at high temperatures) [103].

The state of the art for the top coat is yttria stabilized zirconia, however, there is a continuous search for alternative candidates, motivated by the industry’s demand for more and more performing (and therefore hotter) engines. The main drawback of YSZ is, in fact, its stability at temperature higher than 1200 °C, due to phase transformation with volume increase upon long term exposure (generally greater than 100 h) at high temperatures. Moreover, after long exposure at high temperatures, YSZ tends to sinter and/or to increase its thermal conductivity and is also susceptible to CMAS (calcium magnesium alumino silicates) infiltration [105]. As the service temperature of engines has increased due to the use of novel high-performance materials for TBCs, new mechanisms for failure have evolved. Especially during take-off and landing, gas turbine engines ingest large quantities of air that may contain significant quantities of sand, dust, or ash particles. Due to the high temperatures in the turbine area, the CMAS may reach melting temperature and infiltrate the porosity of the TBC, leading to nearly complete spallation of the top coat. The ratios of the main CMAS constituents, as well as content of impurities (iron oxide, alkaline, and alkaline earth oxides, etc.), modify the melt viscosity and tendency towards crystallization on cooling. CMAS can attack the TBC also by solid state reactions and sintering on the TBC surface. The general mechanism involves melting of the lowest temperature eutectic in the CaO—SiO_2_—Al_2_O_3_ ternary system around 1170 °C, formation of a silicate melt pool, penetration of the porous TBC, and dissolution of the top coat material into the melt. In the case of YSZ coatings, the melt reaches local saturation of zirconia, but not yttria followed by reprecipitation of destabilized zirconia, typically forming small globular grains of zirconia near the TBC surface [106].

The failure mechanism of YSZ APS thermal barrier coatings subjected to CMAS attack was investigated by Čelko et al. [107]. For real applications at the industrial level, the thermal barrier coating candidate should possess, besides the above mentioned prerequisites, also a low residual stress deriving from the fabrication process, to avoid its premature failure by spallation and cracking during service. It is known that when searching for a substitute, one of the main challenges is to overcome the barriers due to restraining forces, such as the speed of the material substitution process, the risks of performance losses, the technological locks due to investments and material qualification costs [7]. In addition, another challenge, before the industrialization step, is the testing of the coating performances in simulated real engine environment, due to the high temperatures employed, the high impact velocity (about 490 m/s), and the size and composition of CMAS.

The difficulty in reproducing the real engine environment, due to the lack of powerful heating and cooling technique associated with the poor heat absorption and conductivity of the ZrO_2_ top coat in the TBC system, and the difficulties in establishing a service-like temperature gradient across the thickness of the TBC system, explain also why only a limited number of studies have been performed on TMF tests of TBC coated alloys [108].

In ref. [109] a laser-rig was shown useful to provide data regarding the ranges of thermal gradients in which TBC/component architectures can operate safely. The experiment was carried out on a benchmark porous APS YSZ TBC, working within the known acceptable maximum temperature envelop conditions of a TBC/substrate system, i.e., T_ysz_ ~1300 °C and T_sub_ ~1000 °C.

In the following, a brief review of some of the most employed materials used for thermal barrier coatings (both single layers and multilayer systems) and of their main fabrication process is reported.

Despite the first TBCs being firstly successfully tested in the mid 1970s at the laboratory scale on a gas turbine engine and entering the revenue service on the vane of a gas turbine engine at the beginning of 1980s, the earliest ceramic coatings investigated for aerospace applications were frit enamels [110]. The first of these frit coatings were developed by the National Advisory Committee for Aeronautics (NACA) and the National Bureau of Standards (NBS). Frit enamels were used in aircraft engines throughout the 1950s. The transition layer between superalloy and enamel coatings ensured the adherence of the coating to substrate and, implicitly, it determined the working life of the parts. One of the most important issues was to create the condition of formation of a continuous chain of -Me-O-Me-O- bonds from bulk substrate till the bulk enamel coating. The phase nature of the transition layer was a mixture of oxides, mainly of Cr_2_O_3_, BaO, and SiO_2_ and of BaCr_2_O_4_ spinel. Despite the good adhesion of the enamel TBC, the working temperature was highly limited by the formation of vitreous phases with lower melting temperature [110].

Later, flame-sprayed Al_2_O_3_/CaO-doped ZrO_2_ ceramic coatings were developed. However, they did not prove to be viable materials for the more advanced thermal barrier applications due to relatively high thermal conductivity of Al_2_O_3_ and its phase transitions, leading to shrinkage and the associated cracking reducing the coating life [110]. In these early ceramic coatings, the bond coat (if present) was nichrome (NiCr) or molybdenum.

In the mid-1970s, NASA introduced a two-layer TBC consisting of a porous atmospheric-pressure plasma-sprayed ZrO_2_-Y_2_O_3_ ceramic over a plasma-sprayed NiCrAlY bond coat, successfully tested on the turbine blades. Y_2_O_3_-stabilized ZrO_2_ was found to be the most suitable material for TBC applications, with an optimum amount of Y_2_O_3_ around 7–8 wt.% (4–4.5 mol.%). This composition offers a high degree of resistance to spallation and excellent thermal stability [111]. The literature is full of reports about YSZ coatings developed as TBCs and fabricated by different processes, mainly EB-PVD and plasma spray, and YSZ is actually still the material of choice for this application [112,113,114,115].

Recent studies reveal a high interest in TBCs based on rare earth oxides (REOs). Rare earth zirconates (RE_2_Zr_2_O_7_) with pyrochlore structure have been frequently used as an alternative to YSZ, due to their thermal conductivity and higher resistance to CMAS attack with respect to YSZ. Dissolution of REOs into the CMAS melt results in the rapid precipitation of apatite (Ca_2_RE_8_(SiO_4_)_6_O_2_) grains. This reaction phase has proven effective in arresting CMAS infiltration in both EB-PVD and APS coating [116,117,118]. In general, they have an ordered fluorite structure with defective anion sites specified as A_2_B_2_O_6_O’, where A comprises rare earth elements, while B consists of a transition metal, most commonly, Zr^4+^ [119].

Rare-earth zirconates, of type Ln_2_Zr_2_O_7_ (where Ln = La, Nd, Sm, Gd, Pr, Ce), have been recently introduced as alternative materials for a new generation ceramic coating for TBCs [120,121]. The melting point of rare earth zirconates is over 2000 °C. They have higher transformation temperatures than those of YSZ and their coefficient of thermal expansion is close to that of YSZ. However, the hardness values of RE_2_Zr_2_O_7_ are lower than YSZ [122,123,124].

In ref. [125], Sm_2_Zr_2_O_7_ coatings obtained by atmospheric plasma spraying presented the same phase as the raw material powder made from coprecipitation. After thermal cycle test for 30 times no change of phase structure was observed in Sm_2_Zr_2_O_7_ coating, but oxidation (led by TGO) was determined which induced to failure of the coating system at the top coat - bond coat interface.

Gd_2_Zr_2_O_7_ (GZO) has attracted particular attention due to the desirable thermo-physical properties, its excellent phase stability and good CMAS resistance [126,127,128,129,130]. 

An analysis of the current status of thermal barrier coatings is reported in ref. [131].

The increasing demand for new functional coatings has been a strong incentive for research, not only towards understanding the fundamentals and technical aspects of film nucleation and growth, but also towards developing new deposition techniques allowing for a better control of the deposition process [132]. EB-PVD, powder flame spraying, plasma thermal spray, cold gas dynamic spray coating, sputtering and CVD (Chemical Vapor Deposition) are techniques suited for creating different types of TBCs [133,134,135,136,137,138,139,140]. Actually, EB-PVD and APS are the most widely used techniques for obtaining TBCs and different multilayer coatings from a large class of different materials. These techniques are able to provide low density coatings with a low thermal conductivity, being generally the thermal conductivity of APS coatings lower. 

In the last decade, advanced vacuum plasma spraying processes, including LPPS (low pressure plasma spraying), PS-PVD (plasma spraying physical vapor deposition) and PS-CVD (plasma spraying chemical vapor deposition) have been proposed for the deposition of TBCs. These processes are characterized by a low pressure in the deposition chamber and gas-phase, rather than liquid-phase deposition [141,142,143]. The basis of PS-PVD is the LPPS technology that has been well established in industry since several years [135].

Table 2 gives the main characteristics of the different coating technologies used for TBCs fabrication.

The EB-PVD process takes place in a high vacuum chamber, ensuring a relatively high deposition rate up to 150 nm/min. The adhesion of the coating may be improved by heating the substrate during the growth process. The EB-PVD process may be successfully used for selective deposition of multi-layered films based on refractory metal, oxides, carbides, nitrides, etc. for the components to be used in extreme conditions. A specific aspect of EB-PVD coatings is related to their columnar microstructure determining the behavior of the coating during its service life [132,144,145].

This structure results in very high yielding strain tolerance, which occurs during thermal cycle changes, but it also reduces the mismatch of thermal expansion between BC and TC, promoting longer lifetime of the TBC system [146]. The control of substrate temperature on coatings adhesion remains a major issue for optimizing the final properties [147,148].

The EB-PVD method is the most expensive industrial coating method manufactured and one of the most complicated [146].

Atmospheric plasma spray APS, sometimes called powdered plasma spray (PPS), is used generally for less fragile elements due to the much lower cost of production. During the APS process, the pulverized raw materials are transported by noble gases, such as Ar, to the APS torch. The heat source is generated using electric arcs. The plasma jet provides thermal and kinetic energy to the material to be deposited, directing it to the desired substrate where it forms the coating. The main APS parameters include current, primary air flow, carrier air flow, powder feed rate, spray distance, and substrate tangential speed [118,146].

Differently, the suspension plasma spray (SPS) process uses very fine suspended liquid particles in the liquid phase to inject the coating material into the plasma torch. Compared to the APS method, SPS is a more complex technique, which requires more training, but which offers improved properties of the obtained coatings [146,149,150].

**Table 2:** Coating technologies for TBCs and main characteristics. Based on data in [151].

Process	Substrate Heating	Deposition Rate	Uniformity for Complex Shapes	Film Density	Morphology
E-beam Evaporation (EB-PVD)	Yes normally	Very high	Poor	Poor	Columnar
APS/SPPS	Not normally	Very high	Poor	Poor	Splat
Sputtering	Yes or No	Low	Poor	Good	Columnar
PS-PVD (atmospheric, plasma spray, jet+ EB-PVD)	Yes normally	Very high	Good	Poor	Columnar
CVD	Yes	Moderate	Good	Good	Columnar
PS-CVD	Yes	Higher than CVD	Good	Poor	Columnar

The deposition process has a strong influence on the micro-structure and morphology of a coated layer. The microstructure of the EB-PVD coatings has a mechanical resistance and a thermal shock resistance, which considerably improves their service life.

Coatings applied by plasma spraying show a very distinct defective and porous structure consisting of a typical splat-like microstructure, generally composed of overlapped lamellae separated by splat boundaries and embedded in a network of microcracks and voids [152,153,154,155].

**Figure 13:**
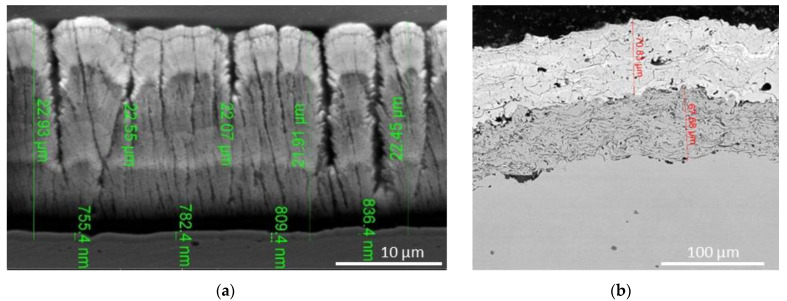
SEM micrographs showing the typical morphology of YSZ coatings deposited on Nimonic 80A substrate: (**a**) columnar structure of EB-PVD coating and (**b**) splat morphology of APS coating.

The characteristic morphology of EB-PVD and PS coatings is shown in Figure 13.

The negative aspect of EB-PVD coatings is the columnar microstructure that causes a higher thermal conductivity compared to that of APS coatings containing micropores reducing the thermal conductivity. Therefore, the microstructure of the coating is an important factor that determines the behavior of the coating over its lifetime.

In addition, apart from film’s microstructure, the thermal radiation transport through the TBC can be regulated also by proper design of the coating, i.e., by using multilayer structures allowing a reduced photon and phonon transport inside the material. Advanced multilayer structures top coats may provide also further reduction of the thermal conductivity and/or an enhancement of the mechanical properties and resistance to erosion, wear, cracking, delamination etc. Multilayer TBC systems consist of an alternate sequence of a low (L) and a high (H) refractive index material (general with quarter wave optical thickness). High reflectance multiple layered ceramic stacks are designed to reflect thermal radiation in the wavelength range of ~0.45–5 μm (the central wavelength range of the reflector depends on the layers’ thickness) and are coupled with a single ceramic layer with low thermal conductivity, a bond coat and the metal substrate. The above mentioned wavelength range is based on the typical operating temperature of ~1700–2000 K in a gas turbine combustion environment [156].

**Figure 14:**
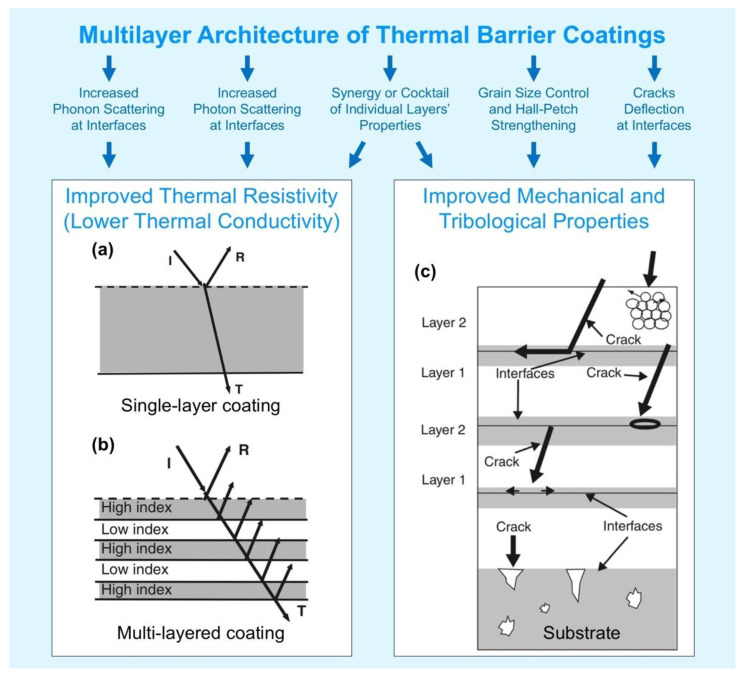
Two approaches of multilayer structures applied in thermal barrier coatings. Low thermal conductivity by reducing the photon transport: single layer structure (**a**) vs. multi-layer, multi-material TBCs structure (**b**). Toughening and strengthening mechanisms in multilayer coatings (**c**). Adapted from figures reprinted from [157,161] with permission from John Wiley & Sons (Copyright © John Wiley & Sons) and from Elsevier (Copyright ©Elsevier), respectively.

Figure 14 compares schematically a single layer and a multilayer TBC architecture.

In contrast to metal alloys (heat transfer occurs mostly by electrons’ movement) usually used in engines manufacturing, main components of TBCs are non-metal and the thermal transport mostly depends on phonon and photon phenomena. Phonon-lattice and phonon-phonon scattering occur on cracks, pores, and point defects (can be driven by dopants) in TBCs, which shorten the mean free path of phonons and decrease the thermal conductivity [49,157]. In addition to the described interactions, phonon-phonon scattering may occur also on the interlayer interfaces in thin multilayer coatings, which was the subject of many studies in the last decades. However, there is still a lack of knowledge in the application (and efficiency) of this phenomenon to the real TBCs [158,159,160].

Photon contribution to the total heat transfer calculation is often underestimated, but can cause a significant effect, especially at elevated temperatures and in near-transparent TBCs, such as partially stabilized zirconia [157]. That is why multilayer coatings with different refractive indexes and densities of individual layers may be considered for further improvement of TBCs, as shown in Figure 14b.

J.M. Kelly et al. [157] predicted by calculations that the thermal radiative properties of the ceramic single-layer 7YSZ can be increased from ∼35% to nearly 100% by using a 7YSZ/Al_2_O_3_ multilayer coating system with layer thickness of 700 nm (YSZ) and 90 nm (Al_2_O_3_). They expected that with such coating, the turbine blade surface temperature would be decreased up to ∼∆180 °C, resulting in 10 times longer lifetime of the component. Additionally, it may be possible to benefit from nanolayered structure in regard to superior creep properties and impact resistance. In their experimental work, they showed it was possible to achieve 73% IR reflectance at 1.85 µm wavelength for multilayer 7YSZ/Al_2_O_3_ coatings (individual layer thickness for 7YSZ was 400 nm and 75–100 nm for Al_2_O_3_ layer). In another study reported by M.P. Schmitt, it was shown that multi-layered t′ low-k/GZO coatings (where t′ low-k is Yb and Gd doped yttria stabilized zirconia ZrO_2_: 2Y_2_O_3_ + 1Yb_2_O_3_ + 1Gd_2_O_3_ (mol.%) and GZO is a rare earth pyrochlore Gd_2_Zr_2_O_7_) can provide lower thermal conductivity than one of these two single-layer coatings, low sintering, and half of the GZO erosion rate [162]. Later, an out-diffusion of Gd from GZO layer under higher temperature and longer term annealing conditions was reported by A.K. Rai et al. [163].

Another research on 7YSZ/Al_2_O_3_ multilayer TBCs was performed by D. Josell et al. [164]. They studied heat transfer through nanoscale multi-layered TBCs at elevated temperatures and tried to evaluate the contribution of interlayer interfaces to the total thermal resistance of the coatings. For 7YSZ/Al_2_O_3_ coating with 6 nm thick individual layers, the two interfaces in each bilayer increase the thermal resistance by 44–100% and, thus, result in a lower thermal conductivity. The latter (effective thermal resistivity 1/K_eff_) strongly depends on the individual layer thickness (d_1_ and d_2_), their thermal conductivity (K_1_ and K_2_), number of interfaces and interface thermal resistance (ITR) as shown by Equation (1) [164]:

1/K_eff_ = (d_1_/K_1_ + d_2_/K_2_ + 2*ρ*)/(d_1_ + d_2_). (1)

The authors suggested that careful selection of materials for multi-layered TBCs is necessary to have both layers with not only low thermal conductivity but also with their values closer to each other than those of Al_2_O_3_ and 7YSZ, so that ITR can add to the performance of the superior material rather than compensate for the deficit caused by incorporation of the inferior. It should be noted that significant values of ITR contribution were observed not for all multilayer specimens examined in this study. Also, the used method had some limitations for ITR calculation for the samples with thick (57 and 370 nm) individual layers. Thus, further research is required to study more precisely the ITR role and the influencing factors. Earlier, K. An et al. [165] also studied Al_2_O_3_/8YSZ TBCs with individual layers from 120 nm to 49 µm and concluded no contribution of interlayer interfaces to thermal resistance (it was on the level of average resulted values from Al_2_O_3_ and 8YSZ). However, it should be noted, that for such thick layers, it may be hard to distinguish ITR contribution (Equation (1)).

M.G. Gok et al. [166] studied multi-layered and functionally graded Gd_2_Zr_2_O_7_/CYSZ TBCs produced in 2, 4, 8, and 12 layers using High Velocity Oxygen Fuel (HVOF) and APS processes to improve the thermal cycling performance of single-layered Gd_2_Zr_2_O_7_. It was possible to achieve relatively lower thermal conductivity for multilayer coatings. The authors assumed that it also could be related to the higher level of porosity and, thus, to increased phonon scattering rates. At the same time, multilayer and functionally graded coatings with thin individual layers demonstrate better thermal cycling and low level or no spallation or microstructural cracks.

In the recent paper by P.G. Lashmi et al. [167], three groups of plasma sprayed multi-layered TBCs were reviewed: Gd_2_Zr_2_O_7_/YSZ, La_2_Zr_2_O_7_/YSZ, and La_2_Ce_2_O_7_/YSZ. The authors identified these systems as the most promising TBCs among the other emerging alternatives to overcome the issues related to single-layer YSZ TBCs such as phase transformation, thermal stability, and thermal cycling life.

Apart from multilayer systems, several multiple layers or composites have been investigated. Ozgurluk and collaborators developed by APS technique a layered TBC system based on YSZ/Ga_2_Zr_2_O_7_ powders obtained by the co-precipitation method. This system has been shown to be effective in preventing the penetration of corrosive salts into the YSZ layer, as demonstrated by the analysis of the FE-SEM cross section of the YSZ/GZO TGO after the corrosion test. Compared to single-layer 8YSZ coatings that showed a thermal cyclic life of 175 cycles at 1100 °C, YSZ/GZO systems have a higher thermal cyclic life of 300 cycles [120].

Using the same APS deposition technique, M. Bahamirian et al. obtained single-layer coatings of nanostructured GZO and double-layer coatings YSZ/nanostructured GZO on Ni-based superalloy (IN738LC) substrates as a topcoat with a CoNiCrAlY bond coat. A better resistance to oxidation was of course noticed in the case of YSZ/Ga_2_Zr_2_O_7_ coatings which could be attributed to prevention from the formation of GdAlO_3_ compounds between the bond and top coat, as well as to the restriction of oxygen penetration. By applying the YSZ layer, which has high fracture toughness, between the Ga_2_Zr_2_O_7_ layer and the CoNiCrAlY layer, it was possible to improve the mechanical properties and prevent the propagation of cracks in the TBC during thermal cycles [168].

The fact that YSZ/Ga_2_Zr_2_O_7_ coatings have a higher heat corrosion resistance than simple coatings has been proven in another study. These superior ceramic coatings were obtained by the EB-PVD method on the CoNiCrAlY bonding layer and were subjected to hot corrosion tests by spreading mixtures of 45% Na_2_SO salt and 55% V_2_O_5_ at intervals of 5 h at 1000 °C [169].

Mahade et al. investigated a multi-layered Gd_2_Zr_2_O_7_/YSZ TBCs fabricated by SPS, which was shown to have a lower thermal conductivity if compared to the single layer YSZ coating, despite the fact that the single layer system had a higher porosity content. The GZ/YSZ TBCs also had a lower thermal diffusivity than the single layer YSZ TBC and a longer thermal cycling life than the single layer YSZ TBC at 1300 °C [116].

The pyrochloride compound, lanthanum zirconate (La_2_Zr_2_O_7_) has specific properties for TBC, and a lower thermal conductivity (1.6 W/(m·K), at 1000 °C) than that of YSZ (2.12 W/(m·K), at 1000 °C), higher hardness, phase stability, high melting point, sintering resistance, thermal stability in a high temperature environment, and a lower oxygen ion diffusivity which protects the bond coat and the substrate from oxidation [118,122,124,170,171]. Compared to single-layer TBC, it has been found that double-layer TBC has a longer lifespan and better performance. Two systems, YSZ and YSZ/La_2_Zr_2_O_7_ obtained by the EB-PVD technique, were exposed to isothermal and thermal cyclic oxidation tests. For the YSZ/ La_2_Zr_2_O_7_ coating, both a longer service life than YSZ TB and better performance than a single YSZ layer were registered [172].

The same finding was made for five other systems YSZ, La_2_Zr_2_O_7_, Gd_2_Zr_2_O_7_, YSZ/La_2_Zr_2_O_7_, and YSZ/Gd_2_Zr_2_O_7_, deposited by EB-PVD technique and exposed to furnace thermal cyclic oxidation tests. As a result of the cycling tests performed, the best performance was presented by the coating system YSZ/Gd_2_Zr_2_O_7_ [173]. By using Al_2_O_3_/La_2_Zr_2_O_7_/YSZ in the design of TBC, two graded coating systems were obtained: a five-layer system (100% Al_2_O_3_)/(75% Al_2_O_3_ + 25% La_2_Zr_2_O_7_)/(25% Al_2_O_3_ + 25% YSZ + 50% La_2_Zr_2_O_7_)/(50% YSZ + 50% La_2_Zr_2_O_7_)/(75% YSZ + 25% La_2_Zr_2_O_7_) and a six-layer system Al_2_O_3_/(75% Al_2_O_3_ + 25% La_2_Zr_2_O_7_)/(25% Al_2_O_3_ + 25% YSZ + 50% La_2_Zr_2_O_7_)/(50% YSZ + 50% La_2_Zr_2_O_7_)/(75% YSZ + 25% La_2_Zr_2_O_7_)/(100% YSZ), using the APS technique. The protection of the metal layer against oxidation is provided by the alumina used directly as a starting layer, because it acts as a barrier to the diffusion of oxygen. A higher resistance to attack of a molten mixture of Na_2_SO_4_ + V_2_O_5_ up to 50 h of treatment was noted for samples coated with YSZ over 75%YSZ + 25%La_2_Zr_2_O_7_, than those coated with 75%YSZ + 25%La_2_Zr_2_O_7_ top coats [174].

Pyrochlorous structure Sm_2_Zr_2_O_7_ has significantly better insulating properties than 8YSZ [175]. Monolayer (Sm_2_Zr_2_O_7_, 8YSZ) and composite (Sm_2_Zr_2_O_7_ + 8YSZ with weight ratios of 75/25, 50/50, and 25/75) TBCs were obtained by atmospheric plasma spraying by Grzegorz Moskal et al. [101]. The authors used as 2-layer TBC reference materials Sm_2_Zr_2_O_7_ and 8YSZ, they studied the influence of interphase limits and determined their role, ensuring the most effective thermal insulation effect for 8YSZ + Sm_2_Zr_2_O_7_ composite coatings. They noted that the volume fraction of the individual phase components and the presence of interfaces between the compounds determine the isolating effect of TBC composites of type Sm_2_Zr_2_O_7_ + 8YSZ. The thermal conductivity decreases thanks to the introduction of a component with a lower thermal conductivity value.

In ref. [176], Celko et al. investigated the durability and damage mechanisms of amorphous and crystalline CMAS-resistant barium-magnesium-aluminum-silicate (BMAS) thermal barrier coatings with high silica content produced by atmospheric plasma spraying on a MAR-M247 alloy. Strain-controlled low-cycle fatigue of another BMAS system consisting of a mullite (Al_6_Si_2_O_13_) + hexacelsian (BaAl_2_Si_2_O_8_) upper layer at a ratio of 70/30 vol.%, a YSZ interlayer, and a CoNiCrAlY BC, sprayed on MAR-M247 alloy was investigated by Šulák et al. [177].

A review of the existing techniques for removing and repairing damaged thermal barrier coatings is presented in ref. [178].

**Figure 15:**
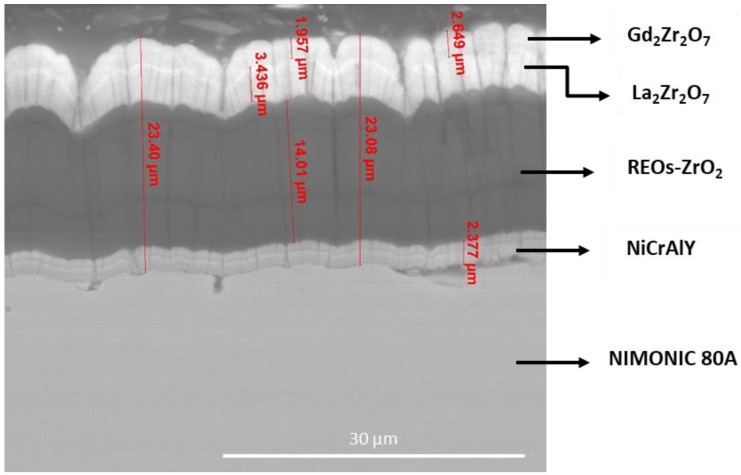
SEM micrograph of a TBC containing REOs-doped Zirconia on NIMONIC 80 superalloy.

Recently, some of the authors of this manuscript fabricated TBCs based on zirconia doped with 8 wt.% multicomponent REOs with the natural composition existing in monazite concentrates [13]. The powders prepared by a hydrothermal method were used to obtain by EB-PVD process multi-layered coatings consisting of YSZ, La_2_Zr_2_O_7_, and Gd_2_Zr_2_O_7_ on Nimonic superalloy, using NiCrAlY bonding layer, as shown in Figure 15.

The results of thermal shock tests show a satisfactory behavior for a number of minimum 150 cycles in the temperature ranges 1200−1300 °C, promising to be a new process that enables the reduction of the extraction and separation costs of these CRMs used as doping elements. Starting from a 6-inch target obtained with the same REOs-doped ZrO_2_ powders composition, 1 µm thick coatings were deposited on NIMONIC 80A by radio frequency sputtering in Ar + O_2_ atmosphere. Preliminary characterizations of these innovative sputtered coatings are still in progress.

LCF performance of Ni-based superalloy coated with YSZ conventional thermal barrier coating have been rarely investigated. As reported by Šulák et al. [96], results on studies on LCF of YSZ coated Ni superalloys are a bit controversial, showing in some case a beneficial [179], an insignificant [180], or a detrimental effect [181] on the fatigue life. In their work, Šulák et al. have investigated the effect of high-temperature (900 °C) LCF performance of a complex TBC-coated and uncoated Inconel 713LC sample. The TC was a mixture of a near eutectic nanocrystalline ceramic made of ZrO_2_, Al_2_O_3_, SiO_2_, and conventional YSZ ceramic in the ratio of 50/50 in wt.%. They found that the TBC enhances the fatigue life in the Coffin–Manson representation and does not decrease the fatigue life in the diagram of the total strain amplitude versus the number of cycles to fracture.

A small survey of the LCF of coated and uncoated Ni alloys is reported in ref. [94].

### 3.2. Surface Treatments and Coatings for Ti-Based Alloys

The major disadvantages of all titanium alloys, especially in aerospace applications, are their poor resistance to abrasive wear, high tendency to seize, high coefficient of friction (COF), and relatively low hardness. In addition, when the working temperature is higher than 600 °C, Ti alloys show a drastic decrease in strength so that they cannot withstand high loads in high temperature environments. Cracking is a notorious issue with Ti. Titanium plates commonly used in the aerospace applications are, in fact, sensitive to fatigue, in particular notch sensitivity, which reduces their strength and consequently leads the material to crack throughout the component. Advanced surface treatments can prevent this issue [182]. Moreover, in view of a wider application of titanium alloys in aerospace engines at elevated temperatures, the threat of oxidation should be prevented. Unlike Ni- based superalloys, Ti-based superalloys lack, in fact, the formation of an Al_2_O_3_ scale and the degradation of Ti-based alloys is characterized by the formation of a rapidly growing scale of rutile TiO_2_ or TiO_2_ + Al_2_O_3_ mixtures and a brittle oxygen-rich sublayer underneath the oxide scale with enhanced attitude to cracking [77,183].

Raluca Pflumm et al. [184] reviewed different coating methods to protect TiAl-based alloy from oxidation. The poor tribological properties of titanium alloys can be improved by specific surface treatments, such as thermal oxidation, plasma nitriding, ion implantation, spraying, laser modification, and plasma electrolytic oxidation (PEO) [185]. In addition, titanium alloys elements are more and more widely joined by adhesive bonding, a technique that constitutes an alternative to traditional joining methods and surface preparation is one of the first stages of the adhesive bonding process, guaranteeing optimal bonding.

We will discuss briefly two of the above-mentioned surface treatments, i.e., laser shock peening, because of its importance in preventing metal alloys fatigue, and plasma electrolytic oxidation for its potential future application in alloys for aerospace. Also, heat resistance coatings grown by conventional PVD, CVD, and spray techniques will be described. Many of the literature data concern Ti-6Al-4V alloy and other (ɑ + β) alloys, the first one as already said, being greatly employed in the aerospace industry.

#### 3.2.1. Surface Treatments of Ti Alloys

##### Plasma Electrolytic Oxidation

PEO, also known as micro-arc oxidation (MAO), is a treatment applied to lightweight metals such as Al, Mg, and Ti to enhance their corrosion and wear resistance properties, or to confer various other functional properties including anti-friction and thermal protection. It combines the control and uniformity of an electrolytic process with an intense local injection of energy (through micro-arcs). PEO is also considered an environmentally friendly and cost-effective coating process with real industrial applications. It is, in fact, generally conducted at room temperature and in a dilute and ecologically safe electrolyte, it requires a short time process, and therefore is less energy consuming in comparison with thermal oxidation or thermochemical processes (nitriding or carburizing) [186,187]. The PEO ceramic coatings growth process depends on several parameters such as duty cycle, process time, current density, frequency, electrolyte, etc. PEO enables the production of high coating thicknesses (over 100 µm), which are less prone to cracking and spallation under high wear stress with respect to conventional PVD and CVD coatings. The plasma enhancement helps to synthesize materials far harder than the amorphous oxides formed by conventional anodizing process [188]. On Ti alloys, a mixture of rutile and anatase titania, silicates, and aluminum titanate coatings may be obtained. These hard surface layers can significantly enhance the wear resistance of the bare substrate. Moreover, to improve specific coating performances, nanocomposite coatings may be obtained by adding various insoluble nanoparticles into the electrolyte. In this process, the type and concentration of nanoparticles directly affect the properties of the coatings. The most commonly added particles are various oxides including TiO_2_, Al_2_O_3_, SiO_2_, CeO_2_, ZrO_2_, etc. [189]. The particles can achieve either reactive or inert incorporation into the coating while imparting excellent properties to the coating. PEO coatings are capable of withstanding higher wear and of providing more resistance to cracking. Specific benefits include a reduction of the coefficient of friction from ∼0.8 to ∼0.4 against steel, which would lessen the damage from galling [182]. Li et al. reported a slight increase in wear resistance of the MAO coating fabricated in Al_2_O_3_ added silicate-based electrolyte on Ti-6Al-4V alloy [190]. In another paper by Ding et al., different contents of Al_2_O_3_ additive were introduced into a NaAlO_2_ basic electrolyte with the aim of improving the high-temperature properties of PEO ceramic coatings on Ti_2_AlNb alloy [191]. Excellent oxidation resistance of ceramic coatings on Ti_2_AlNb substrate was mainly attributed to the formation of the Al-containing oxide layer. Moreover, an enhancement in wear resistance at room temperature was also observed. Li et al. [192] reported that the concentration of ZrO_2_ in a phosphate-based electrolyte plays a crucial role in the wear resistance of the MAO coatings fabricated on Ti-6Al-4V alloy.

However, process repeatability is an issue that needs to be looked at before it can be introduced on an industrial scale, requiring further efforts for optimization of the parameters and conditions concerning proper electrolyte candidates, time of the treatment, and final results with mechanical and surface properties on request [76]. In most of the sectors, PEO processes are actually in a transition phase from research to industrial application, mainly focused on the corrosion and wear protection of light alloys. The behavior of PEO coatings for aerospace application is still at the laboratory scale study and some of the research performed up to now demonstrates that PEO coatings on Ti-6Al-4V may not perform well under intense wear, but have great potential for aerospace components such as fasteners and disks that sustain low but prolonged wear [193].

##### Laser Shock Peening

Laser shock peening is a post-processing technology that utilizes a high-power laser working in pulse mode. The aim of LSP applied to SLM fabricated parts is to produce compressive residual stresses (CRS), which influences with a significant increase in fatigue resistance, through the cancellation of natural tensile residual stresses (TRS) resulting from the solidification processes. As the last deposited layer cools, it shrinks, but this shrinking is limited by the already solidified underlying material [194]. The increased in-depth compressive residual stresses, apart from improving fatigue performance of materials, can also control the development and growth of surface cracks [195,196,197].

In the aerospace industry, LSP can be used to treat many products, such as turbine blades and rotor components as demonstrated by several patents since 1996 [198,199], LSF was also used to treat the leading edges of turbine fan blades in an F101-GE-102 turbine using LSP for the Rockwell B-1B bomber, which enhanced fan blade durability and resistance against foreign objects damage (FOD). During LSP, surface texture of the part is not affected as it is if the conventional shock peening (SP) would have been used. Moreover, CRS occur at higher depths [200]. The basic principle of LSP is similar to SP by the application of shock wave. In LSP, the shock wave is induced by plasma pressure, deforming the surface layer, influencing significant grain refinement [201]. The plasma pressure is focused and concentrated into the material by the application of transparent external confinement, placed on top of the workpiece. There are several types of confinement, the most significant being liquid and solid, water and glass or polymer, respectively [202,203,204]. Besides fatigue, which is improved mostly, other base material inherent properties can be enhanced by applying LSP, such as wear and corrosion resistance, also not less important from the engineering and application point of view [205].

LSP parameters used in Ti-alloy treatment published in some recent works are given in Table 3. It can be seen that as in LSP of other materials, NdYAG laser operating at 1064 nm wavelength and Al tape or foil, as well as water confinement, are standard. Pulse durations are between 10 and 20 ns and laser spots between 2.4 and 4 mm are utilized. Pulse energies varying generally between 0.1 and 100 J are sufficient to produce beneficial effects in the material surface. Zhou et al. [202] found that LSP influences the CRS induction, which is the highest in the surface layer. CRS and microhardness values gradually drop as the depth is higher. At 0.8 mm, the residual stresses become tensile in nature, as in the base Ti-64 (Ti-6Al-4V) alloy, as well as microhardness values. Multiple LSP impacts resulted in a more pronounced CRS and higher microhardness values. These results were correlated to microstructures, where no distinguishable phase transition was established, rather, dislocation multiplication and grain refinement were induced. Moreover, nano-grains were formed in the shocked surface, with more homogenous nanograins obtained after multiple LSP impacts. A similar trend in microhardness was found in the work by Lan et al. [206]. Furthermore, although the LSP depth achieved was 0.5 mm, tensile strength and yield strength were increased, at the loss of elongation.

The work of Yang et al. [207] investigated the effect of LSP on Ti-64 subjected to foreign object damage of the jet engine compressor blades, using a 3 mm diameter 62–64 HRC (Hardness Rockwell C) steel ball fired from a gas gun, as an initiation for the crack in the specimen. These cracks were propagated by a subsequent dynamic loading. It was found that LSP treatment increases the fatigue resistance between 94 and 169% in relation to the untreated specimen.

In Cao et al. [208], Ti-64-based alloys were coated with TiN and LSP treated and subsequently coated with TiN. It was shown that the specimens pre-treated with LSP exhibited higher CRS and significantly increased fatigue strength. Equally significant, there was a notable increase in TiN coating adhesion, arguably the most important coating property.

Square laser spots, otherwise more suitable for a more effective covering of the compressor blade surface, were applied in the study by Zou et al. [209]. The results suggest that surface roughness, important for the suitability of the compressor blade is not significantly affected by LSP. In addition, fatigue life of the specimens was improved, due to the existence of CRS and refined grains: high density dislocations in β phase, and deformed twinning and nano-grains in α phase of the Ti-17-based alloy. Similar findings were reported by Huang et al. [210] and Luo et al. [211].

**Table 3:** Alloys and LSP parameters applied in various published works.

**Alloy**	**Laser** **Type**	**Wavelength,** **nm**	**Pulse** **Duration, ns**	**Pulse** **Energy, J**	**Laser** **Spot, mm**	**Absorption** **Layer**	**Confinement**	**Source**
Ti-64	NdYAG	1064	10	7.9	Ø 3	Al tape 0.12 mm	Water 3 mm	[202]
Ti-64	NdYAG	1064	12	11.89 GW/cm^2^	Ø 2.5			[206]
Ti-64	-	1064	20	4	Ø 2.4	Al foil 0.1 mm	Water 1 mm	[208]
Ti-64	NdYAG	1064	10	7.6	Ø 3	Al foil 0.1 mm	Water	[207]
Ti-17	NdYAG	1064	15	-	3.97 × 3.93	Al foil	Water	[209]
Ti-17	NdYAG	1064	10	7	Ø 3		Water 2 mm	[210]
Ti-17	-	-	20	3.4 GW/cm^2^	Ø 2.5	Al foil	Water	[212]
Ti-11	-	1064	20	6	Ø 3	Metallic foil 0.1 mm	Water 1 mm	[211]

#### 3.2.2. Coatings for Ti-Based Alloys

The most important challenge in the development of a coating for titanium alloys is the preservation of their properties over time in corrosive environments and relatively high temperatures. Without any surface protection, Ti alloys can undergo the formation of a rapidly growing scale of non-protective TiO_2_ on their surface. The major strategy against this behavior is favoring the development of protective oxide layers, like α-Al_2_O_3_, presenting long-term stability on the alloys surface during service, so to prevent further deleterious oxidation of the alloy. Besides oxidation problems, the interaction of the alloy with nitrogen in the atmosphere can also lead to the formation of nitride sublayers, like TiN and Ti_2_AlN, at the interface between the alloy and the top oxide scale, interrupting the formation of a protective alumina scale and thus affecting the oxidation resistance of the alloy [213]. For these reasons, proper surface coatings are essential to avoid or retard the occurrence of these phenomena on Ti-based alloys. To this purpose, several coating materials can be considered for Ti alloys protection, like oxides, different kinds of metallic systems, and multi-layered coatings. The different types are described in the sections below.

##### Oxide Coatings

One of the simplest way to provide some protection on Ti alloys is the direct deposition of an α-Al_2_O_3_ protective layer on their surface, guaranteeing the reduction of the alloy’s oxidation rate [214]. In addition, an Al interlayer between the alloy surface and the alumina layer can act as a source for the formation of the oxide layer during the high temperature oxidation and as a barrier layer for Ti outward diffusion, thus favoring the formation of a Ti-Al intermetallic layer that improves the coating adhesion [215].

A similar approach was used in a recent work by H. Lin et al. [216], where a metallic Al-Y interlayer was placed between the TiAl alloy and the Al_2_O_3_-Y_2_O_3_ coating to compensate for the element consumption in the oxide layer. In such coating, different oxides layers were formed during oxidation operation (Al_2_O_3_, Y_2_O_3_, and mixed YAG), with the Y_2_O_3_ being more stable than the Al_2_O_3_, and with the formation of a titanium aluminide TiAl_3_ layer helping to suppress the outward diffusion of Ti from the alloys.

A glass-ceramic coating was applied on Ti-6Al-4V alloy for protection against oxidation at 800 °C. The titanium alloy is effectively protected by the glass-ceramic coating, of which the oxidation develops at constant rate. After oxidation, both the weight gain and oxygen ingress into the Ti–6Al–4V alloy substrate are negligible [217].

Improvement of oxidation and hot corrosion resistance of TiAl alloys can be achieved by the use of more complex glass-ceramic coatings constituted by a mixture of several different oxides (e.g., Al_2_O_3_, SiO_2_, MgO, BaO, ZnO, ZrO_2_, CaO, etc.), like in enamel coatings [218,219]. In the work by Tang et al. [218], an enamel coating on TiAl was shown to be more effective than a simple Al_2_O_3_ coating during cyclic thermal oxidation, thanks to adhesion and absence of cracks or spallation effects in the enamel coating with respect to the less resistant simple alumina coating. Besides the increased protection of the TiAl alloys against high temperature oxidation, enamel coatings can also provide hot corrosion resistance in molten salts by blocking the migration of corrosive ions to the alloy surface [220], even if at longer exposures the oxides components undergo dissolution, causing the coating degradation.

Due to the low cost, easy operation, and excellent thermal stability, sol–gel-based oxide coatings have been also employed to improve the oxidation resistance for TiAl alloys [221].

##### Aluminide and Chromizing Metallic Coatings

Instead of the direct deposition of an alumina coating on the alloy surface, another simple method to obtain a protective coating consists in aluminizing the surface, forming a titanium aluminide layer, so to get its oxidization under the high temperature operations. However, after oxidation, cracks in the TiAl_3_ layer and consequentially pitting corrosion can happen [222,223].

Another intuitive way is the chrome plating of the alloy surface. During oxidation, the chromium plating coating provides Cr atoms which together with Ti and Al diffuse outwards to form multilayer oxide films, which prevent the diffusion of oxygen [224]. Double-glow plasma surface chromising was performed on Ti–6Al–4V alloys by Wei et al. [222,224], and the isothermal oxidation behavior of Ti–6Al–4V alloy was investigated at 650, 750, and 850 °C. During oxidation, Cr, Ti, and Al diffused outward to form multi-layer oxide films, which prevented the diffusion of oxygen.

The first studies aiming at increasing the operation temperature of alloys for the Ti-Al system up to 800–900 °C concerned conventional diffusion aluminides coatings. Aluminide coatings have been widely studied as protective materials on titanium alloys. Several elements, like Si, Y, Cr, Pt, Ce, etc., have been tested in intermetallic aluminide coatings to improve the high temperature resistance of the alloys by promoting the formation of protective oxide scales (Al_2_O_3_, SiO_2_, Cr_2_O_3_, YAlO_3_, etc.) and blocking the interdiffusion phenomena.

Cr-Al and Ni-Al coatings have been applied on TiAl alloys to increase the oxidation resistance [225,226]. In a very recent work by Guo et al. [226], Ni-Cr-Al monolayer and Ni-Al/Cr-Al bilayer coatings were synthesized on the Ti–6Al–4V alloy, showing the occurrence of some cracking effect at the interface between the monolayer coating and the alloy surface, while no significant defects were observed in the bilayer structure, which resulted in a better oxidation resistance.

Fabrication of Si-Al, Al-Y, and Si-Al-Y layers on TiAl alloys have been shown to produce a series of different intermetallic layers (e.g., aluminide and silicide) and oxide layers, increasing the oxidation resistance of the coated alloys [227,228,229,230,231,232]. In particular, J. Xiang et al. [232] coated a Ti alloy by means of Si-Y co-deposition and aluminization, which, due to inward diffusion of Al atoms and outward diffusion of Ti and Nb atoms, gave rise to the formation of different mixed intermetallic layers and promoted the preferential development of Al_2_O_3_ on the surface in spite of the less resistant TiO_2_ oxide. In a work by K. Bobzin et al. [230], different coatings based on Si-Al and Si-Al-Y were deposited and tested on TiAl, showing an enhanced oxidation resistance thanks to Si and Y addition, promoting the formation of α-Al_2_O_3_, YAlO_3_, and a continuous intermetallic scale of TiSi, with consequent suppression of inward oxidation and outward Ti diffusion.

**Figure 16:**
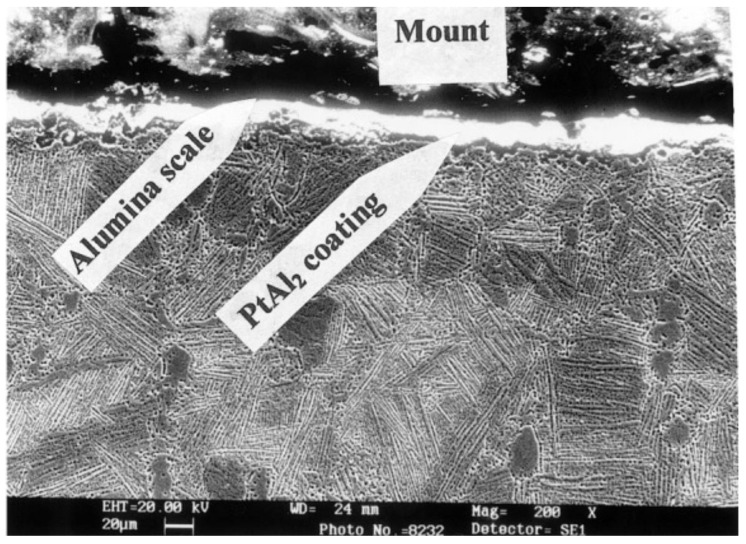
SEM micrograph of IMI 834 alloy showing the presence of an alumina scale on platinum aluminide coating after oxidation for 400 h in air at 800 °C. Reprinted from [233] with permission from Elsevier.

Platinum aluminide coatings seem to protect the titanium alloys from oxidation as well as from hot corrosion, preventing the formation of the alpha phase. In ref. [233], Gurrappa et al. deposited an intermetallic platinum aluminide on IMI 834 alloy by combing electrodeposition and pack aluminizing techniques (Figure 16). They observed an excellent oxidation resistance of the Ti alloys up to 800 °C, thanks to Pt, which effectively promotes continuous alumina scale formation during high temperature exposure.

A PtAl-based coating on TiAl alloy has been applied also in conjunction with a 7YSZ top coat, as reported in [234].

##### Ti-Al-X Metallic Coating Systems

The inclusion of Ti in thermal resistant intermetallic coatings can help increase the compatibility with the underlying Ti-based alloy interface and approaching the thermal expansion coefficients of the different materials, so to prevent possible coating failure due to poor adhesion between the coating and the alloy. Among these, Ti-Al-Si and Ti-Al-Cr systems have been applied to improve the high temperature oxidation resistance of Ti-Al alloys [235,236,237]. In particular, Ti-Al-Cr coatings provide excellent oxidation resistance at 750 °C [238] for up to several thousand hours. These coatings have good resistance to temperatures between 750 and 900 °C. The biphasic microstructure of the coating with the Laves phase [239] is the most resistant to oxidation. At higher temperatures (950 °C), a strong interdiffusion between coating and substrate material occurs by inducing a less thick alumina oxide layer. Once this is broken, the titanium oxide forms rapidly and controls the growth rate of the oxide (breakaway oxidation): the oxide scale consists of protective alumina (Al_2_O_3_) and fast-growing titania (TiO_2_). Moderate Nb addition in Ti-Al-based coatings, like Ti-Al-Nb and Ti-Al-Nb-Si on TiAl alloys [240], has been found to improve the plasticity, oxidation resistance, and wear resistance of the coating.

Quaternary intermetallic coatings like TiCrAlY and TiCrAlZr have been also investigated for oxidation protection of titanium alloys [241,242,243]. TiCrAlY resulted in good adhesion to the alloy and oxidation protection thanks to the formation of Al_2_O_3_ scale, but oxidation degradation was found at increasing temperature due to increasing formation of TiO_2_ and simultaneous decreasing of Al_2_O_3_ and Cr_2_O_3_. TiCrAlY and TiCrAlZr coatings were tested by N. Laska et al. [243] on two different kinds of TiAl alloys, showing good oxidation protection of both coatings, but with significantly different behavior of the two coatings on the different alloys, due to different cracking effects, elemental interdiffusion, and depletion.

##### MCrAlY Metallic Coating Systems

In MCrAlY systems, where M represents the presence of one or more additional metal elements, several elemental combinations have been reported as effective coatings on TiAl-based alloys. The TiCrAlY coating mentioned above is already an example of such kind of coatings. Besides this, NiCrAlY and CoCrAlY coatings are widely investigated metallic systems acting as thermal barrier coatings for TiAl-based alloys. The MCrAlY type coatings are characterized by high reactivity with the TiAl substrate, leading to diffusion layers comprising brittle phases such as AlNi_2_Ti in the case of NiCrAlY coating or AlCo_2_Ti in the case of CoCrAlY. Tang et al. [244] investigated the effect of MCrAIY overlay coatings on oxidation resistance of TiAl intermetallics. They found that at 900 and 1000 °C, TiAl alloys exhibited poor oxidation resistance due to the formation of mixed Al_2_O_3_ + TiO_2_ scale. CoCrAlY and NiCrAlY coatings were effective in decreasing the oxidation rate of TiAl alloy due to the formation of the protective Al_2_O_3_ scale, even though TiO_2_ appeared on both of CoCrAlY and NiCrAlY coatings, which resulted in the acceleration of the oxidation rate at 1000 °C.

MCrAlY systems, however, can present problems related to interdiffusion or formation of unfavorable TiO_2_ which, as already said, has an enhanced attitude to cracking. For this reason, NiCoCrAlY coatings have been applied on TiAl alloys together with the insertion of diffusion barrier interlayers like Al_2_O_3_/Al and Ni-Re, which are reported to suppress these phenomena, thus improving the temperature resistance of the coating [245,246]. Diffusion barriers of Al_2_O_3_ of various thicknesses have been fabricated by filtered arc ion plating between the NiCrAlY coating and the O-Ti_2_AlNb alloy by Li et al. [247]. The authors found that, without a diffusion barrier, interdiffusion between the NiCrAlY coating and the Ti_2_AlNb substrate was very serious and resulted in fast coating degradation and serious oxidation, while interdiffusion and interfacial reaction were effectively suppressed by using an Al_2_O_3_ diffusion barrier. Improved oxidation resistance of the multilayer coating was observed for thicker diffusion barriers.

The performance of NiCoCrAlY coatings can be further modified by the suitable addition of refractory metals, such as Ta or Mo [248,249,250]. In particular, in the work by X. Gong et al. [250], a 2 wt.% Mo addition in a NiCoCrAlY coating resulted beneficial in improving the coating adhesion on the TiAl alloy and inhibiting the interdiffusion, but slightly decreased the oxidation resistance of the coating due to MoO_3_ formation. The same research group [249] reported a similar behavior when the NiCoCrAlY coating was modified with a 4.5 wt.% Ta addition, which increased the coating adhesion and influenced the interdiffusion mechanisms while adversely reducing the oxidation resistance.

As already discussed in the section about Ni-based alloys coatings, multilayer systems can tailor and improve the coating performances. Many coatings, including combination of intermetallics and nitrides have been proposed. Additionally, the thermal barrier characteristics of the intermetallic MCrAlY materials can be further improved when used as bond coats in combination with top coats based on YSZ, as already seen for Ni superalloys.

##### Nitrides and Nitride-Based Multilayer Coatings

Nitride coatings are tested as protective layers on Ti-based alloys, but one of their drawbacks is the formation of penetrating cracks in some areas of the coatings during high temperature operation, which can act as interdiffusion paths of oxygen and Ti and cause severe oxidation. The amount of penetrating cracks increases with the increasing oxidation time. This characteristic of nitride coatings limits their practical application as protective coatings against long term high temperature oxidation [251].

In ref. [252], the authors showed performances of PVD TiN/Ti coatings as multifunctional erosion and corrosion resistant coatings for compressor blades of aircraft engines in harsh environment. The obvious passive film appeared after 960 h salt spray corrosion.

Additionally, Bonu et al. [253] investigated solid particle erosion (SPE) resistant ultra-thin Ti/TiN multilayer coatings with porous and dense energy absorbing (Ti) layers on Ti-6Al-4V substrates. The higher energy absorption property of porous Ti was successfully utilized in resisting the SPE. Finite element simulations and experimental data showed better performance of the coating with porous Ti layers compared to the coating with dense Ti layers at higher erosion speeds (50 to 100 m/s).

Increased chromium content generally reduces the corrosion rate and provides good erosion resistance, improving the chemical stability. Coating hardness decreases with the increase in Cr content, while the corrosion resistance is governed by the coating thickness [254].

Novel CrAlYN/CrN nanoscale multilayer PVD coatings produced by the combined high power impulse magnetron sputtering/unbalanced magnetron sputtering technique showed excellent potential for reliable protection of γ-TiAl alloys against wear and aggressive environmental attack at 750 °C [255].

In ref. [256], the performances of TiBN/AlCrYN multilayer coatings at high temperature (≤900 °C) are shown. Resistance to oxidation is linked to the formation of the dense layer of aluminum oxide which effectively hinders the diffusion of the underlying elements. As the period of the multilayer decreases, the oxidation resistance of the coating increases because the diffusion blocking role of the interfaces is emphasized.

**Figure 17:**
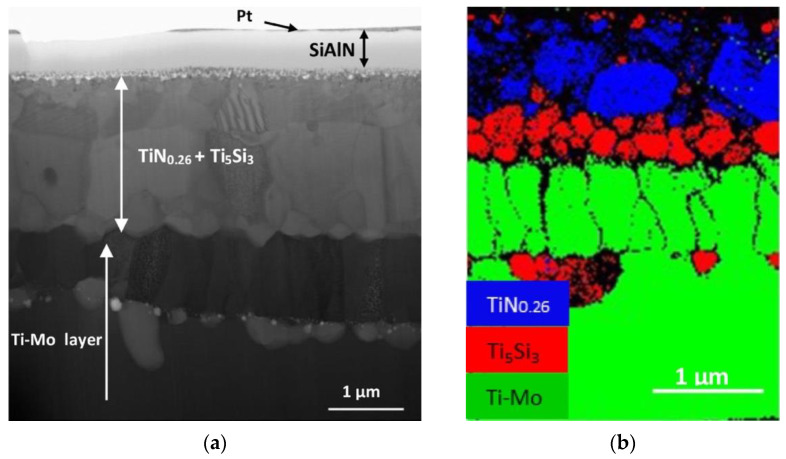
Microstructure and element distribution for cyclic oxidized samples: (**a**) Cross-sectional STEM image of SiAlN/Mo coating on Ti after oxidation in air at 800 °C for 100 h. (**b**) TKD (transmission Kikuchi diffraction) phase map of cross-sectional SiAlN/Mo coating on Ti after oxidation in air at 800 °C for 100 h. Reprinted from [183] under an open access CC BY 4.0 license.

Bilayer SiAlN coatings (1.2 mm thick) with a Mo interlayer (300 nm) (Figure 17) were deposited very recently by magnetron sputtering on Ti and Ti-6Al-4V by Gao et al. [183]. The interdiffusion layer between Ti and Mo, and inter-reaction between Ti and Si_3_N_4_ from the SiAlN coating at the interface during thermal exposure, allowed highly conformable coating with high oxidation resistance and resistant to spalling during thermal cycling.

Often the nitride coatings are able to implement the protection of the components from erosion due to the ingestion of particles [257]. Aggregated cubic boron nitride grains exhibit micro-wear and constant multi-wear properties which are the reason for the improvement of the grinding performance of titanium alloy blades for aeronautical applications [258].

##### YSZ-Based Thermal Barrier Coatings

Thermal barrier coatings (TBCs) similar to those already described for Ni-based superalloys are applicable to Ti-based alloys as well. Many of these use YSZ as top coat, while different bond coat materials are used, as described later. TBCs are necessary to exploit the full potential of gamma titanium aluminides and other Ti-based alloys at moderately elevated temperatures. In TBCs on γ-TiAl alloys, fairly thick oxide scale (20–30 μm), much thicker than the alumina scales (about 7μm) in nickel-based superalloys, can be formed without failure [259]. However, yet no TBC coating system tested so far has proven sufficient performance for a long-term use in automotive and aerospace applications.

Coatings made of CoNiCrAlY (100 µm) metallic bond coat and ceramic YSZ top coat (300 µm) have been studied to improve the high temperature oxidation resistance of TiAl-based alloys [260,261]. Additionally, Zeng et al. deposited thermal barrier coatings with a typical 8YSZ ceramic top coat and CoNiCrAlY bond coat on Ti-6Al-4V by air plasma spraying. They found that the thermal shock life of the TBC on the titanium alloy strongly depends on thermal shock temperature. Elemental diffusion has great effect on acceleration of the samples failure, especially for samples at 1000 °C, because of the allotropic transformation of Ti alloys from 900 to 980 °C, making diffusion much severe [262].

Similarly, highly adherent TBCs made of a NiCrAlY bond coat and a YSZ top coat, together with a thin Ti adhesion layer between the substrate and the bond coat, have been tested on the Ti-6Al-4V alloy [140]. Titanium aluminide TiAl_3_ or other intermetallic materials like Ti-Al-Cr-Zr [263,264] have been also investigated as bond coats for YSZ top coats, with the aim to improve the high-temperature oxidation protection of Ti alloys and the adhesion of the thermal barrier coatings. However, degradation and formation of oxide layers at the interfaces, associated with cracking and delamination effects, have been observed at high temperature due to interdiffusion phenomena between the alloy and the coatings, thus indicating the need of additional diffusion barrier layers [261].

Freitas et al. [265] investigated the creep behavior of a Ti-6Al-4V alloy coated by a plasma spray TBC. The top coat was YSZ and the bond coat also in this case was NiCrAlY. The authors found an increase of the oxidation resistance due to the coating and from their creep rate analysis, they found that the dominant creep mechanism in all cases was the dislocation climb.

In addition, Zhou et al. used 8 wt.% yttria stabilized zirconia air plasma sprayed coatings on Ti-6.6Al-3.61Mo-1.69Zr-0.28Si alloys and investigated their microstructures and mechanical properties. Although they observed no change in the microstructure of the titanium alloy after plasma spraying, nor evident interaction nor atomic diffusion at the bond coat/substrate interface, they observed a thin layer of plastic deformation zone in the substrate beneath the bond coat/substrate interface. Moreover, they observed that the adhesive strength of the TBC decreases with increasing the thickness of as-sprayed ceramic coatings, which is attributed to the residual thermal stresses induced due to the temperature gradient and the mismatch of thermal expansion coefficient during thermal spraying [266].

**Figure 18:**
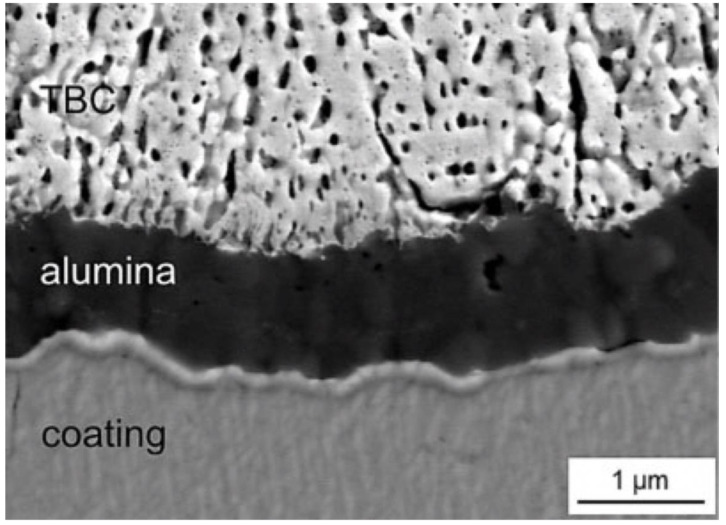
Scanning electron micrograph of a Ti-45Al-8Nb specimen coated with TiAl_3_ aluminide and TBC, which was oxidized at 900 °C in air for 1000 cycles. Reprinted from [267] with permission from John Wiley & Sons. Copyright © John Wiley & Sons.

Braun et al. [267] investigated the performance of thermal barrier coatings on Ti-45Al-8Nb alloys. The top coat was EB-PVD 7% YSZ, while four types of bond coat layers were tested: Al_2_O_3_, TiAl-CrYN, TiAl_3_, and TiAl_2_. Thermal barrier coatings revealed excellent adhesion on oxide scales consisting predominantly of alumina and the failure of the thermal barrier coatings was associated with cracking of the oxide scale in the porous titania-rich layer. The TiAl_3_ aluminide coating provided an excellent oxidation protection associated with the formation of a continuous alumina surface layer (Figure 18).

#### 3.2.3. General considerations

The different coating materials and structures analyzed above represent various solutions that could result in effective protection of Ti alloys for high temperature aircraft components but still need to be improved.

At the same time, it is important to underline how a single surface treatment (like e.g., a coating) could be insufficient to achieve the desired performance in high temperature Ti alloys. Indeed, as pointed out in the recent review from Zhang et al. [268], “*incorporation of different surface modification technologies with high-performance modified layers may be the mainstream of surface modifications for Ti and Ti alloys (and other metals as well)*, *which is striving for high performance and broad applications”.*

We have to consider, in addition, that whatever the surface treatment and/or the coating technology, they add an increase in the cost of the product itself. Cost becomes significantly lower when a mature and industrially available technology is used. However, the durability advantage and the allowable increase in service temperature provided by the use of environmentally resistant coatings will pay off the cost of the coating quite quickly. Long term stability of the coating during surface modification of titanium alloys and titanium aluminides is the main issue. It is therefore essential to gain information about the long-term stability, the high temperature tribological characteristics and the fatigue resistance of coated Ti alloys for their practical industrial exploitation.

Recently, Zhang et al. compared the LCF performance of a selective laser melted Ti–6Al-4V alloy with a wrought Ti–6Al-4V one and found a better LCF performance at low strain amplitudes but a worse LCF performance at high strain amplitudes [269]. The high-cycle fatigue (HCF) of a Ti-6Al-2Sn-2Zr-3Mo-1Cr-2Nb-0.1Si alloy was investigated recently by Tan et al., who studied the influence of microstructure on slip irreversibility and crack initiation during HCF [270].

The fatigue behavior of coated Ti-6Al-4V and GammaMet PX alloys was recently investigated by Zhu et al. In their study, they examined multicomponent TiAlCrTaSiN coatings, and found that coating compositions and especially processing improvements would be needed for Ti alloys coatings before they could be used in service under highly loaded fatigue and oxidation conditions [271].

According to Dai et al. [251], future efforts should be concentrated on the deployment of more reliable alloys employing REEs as alloying elements and on the development of reliable heat resistance coatings which not only will guarantee the oxidation resistance at high temperature but will also preserve the high temperature tribological properties of the alloys.

## 4. Conclusions and Outlooks

This work provides a survey of Ni-based superalloys, Ti-based alloys, coatings, and surface treatments currently used for aerospace engine components. The aim of the work is to raise awareness on the critical raw material content in substrate alloys and coatings and suggest some possible solutions for CRMs saving. Sustainable alternative materials and processes targeted at reducing critical raw element consumption and negative environmental impacts (CO_2_ and NO_x_ emissions, chemicals), while preserving engines’ fuel efficiency are analyzed. A few mentions about the strong revolutionizing potentiality of additive manufacturing processes and machine learning methods for aerospace industry progress are given.

The evolution of Ni-based superalloys used for the hottest engine components allowing them to face the continuous increase of service temperatures has been described. In SX 2nd and 3rd generations Ni superalloys, Re is added to enhance the mechanical behavior and the creep resistance, while addition of Ru in fourth generation alloys guarantees superior high temperature creep and thermo-mechanical fatigue resistance. A reduced resistance against oxidation is observed in fourth and fifth generations due to the high content of Ru, Mo, and Re refractory metals. Sixth generation TMS-238 alloys has been therefore developed by finely tuning the alloying elements in order to enhance the oxidation-resistance at high temperature and improve the creep strength with respect to fifth generation superalloys. Summarizing, in case of Ni-based superalloys components, one big threat is the supply risks of many critical or rare elements contained in the new generation alloys, and the search for high resistance thermal barrier coatings able to allow the substrate to withstand the ever increasing service temperatures of aircraft engines, thus preserving it from failure and indirectly saving its CRMs content.

Recent trends in TBCs development for Ni superalloys consist in replacing YSZ with rare earth zirconates with pyrochlore structure, due to their lower thermal conductivity and higher resistance to CMAS attack. Some preliminary results on mixed REOs-doped zirconia coatings fabricated by EB-PVD by some of the authors of this manuscript in the frame of the EU MONAMIX project are also discussed. The mixed rare earth oxides were extracted directly from monazite concentrate minerals, therefore avoiding complex purification and separation steps of individual lanthanides, thus providing a greener route for doping zirconium oxide. This novel recipe for ZrO_2_-based TBCs, if optimized, may lead to better materials with lower costs and lower environmental impact, for application as TBC for both Ni-based superalloys and Ti-based alloys.

Partial replacement of Ni-superalloys by Ti-alloys, intermetallics, CMCs, and HEAs for improved performances and weight reduction is discussed.

One of the most significant applications of Ti is in lightweight high-strength alloys for aeronautics, space, and defence, where its light weight results in better performance with lower fuel consumption.

Today, the aerospace industry is one of the world’s largest purchasers of titanium and titanium alloys, and increasing Ti use in aerospace and other sectors poses it at supply risk for the EU industry and motivates its ingress in the CRMs list released in 2020. Like Ni-based alloys, also Ti-based alloys contain CRMs as strengthening elements.

A survey of emerging surface treatments and coatings for Ti-based alloys has been given, analyzing their performances in view of a larger potential application of Ti-based alloys as Ni-based alloys substitutes in LP turbine blades. Focus on two surface treatments of Ti alloys, such as laser shock peening and plasma electrolytic oxidation has been given. Additionally, several different coating solutions, tested on Ti-alloys so far, have been reported together with their advantages and limitations: single, mixed and multi-layered oxide coatings; metallic coatings based on aluminide and chromizing layers, Ti-Al-X compounds, and MCrAlY compounds; nitride-based coatings; YSZ-based coatings. Through their use, it is possible to increase the high temperature resistance of Ti-alloys by improving the oxidation resistance and preventing interdiffusion effects, which are among the most important deleterious phenomena in high temperature operation. To overcome the failure of Ti-alloys at high temperature and high loads, further work is still required for assessing the long-term stability, the high temperature tribological characteristics, and the fatigue resistance of coated Ti-based alloys for their industrial exploitation.

In both considered cases (Ni-based and Ti-based alloys), YSZ-based coatings and novel thermal barrier coating solutions are recognized as a key factor for preserving substrate alloys at high temperature for the development of aircraft engines with improved fuel efficiency, reduced environmental impact, and reduced noise generation. The possibility of repairing damaged components is now being developed through new feasible laser processing methods such as laser additive manufacturing, providing a further possibility of CRMs saving in the substrate alloys.

Many of the coating solutions presented throughout this paper contain critical raw materials as well. However, their proper optimization and continuous recent advances allow the effective protection of the underlying bulk alloys, with consequent increased lifetime and operational performance of the coated components, thus effectively helping in saving CRMs and reducing environmental impact. Most importantly, it appears evident how an integrated combination of different aspects together (modelling, evolution of base alloy materials, addition of surface coatings, and use of other surface treatments) is the key strategy to efficiently mitigate the issues related to the use of these alloys under severe operating conditions.

## Figures and Tables

**Table 1:** Nominal chemical compositions, wt.%, of Ni-based alloys. Adapted from [42].

Alloy	Cr	Co	Mo	Fe	Al	Ti	Ru	Mn	Ta	Re	Hf	C	B	W	Nb	Y	Zr	Other
Conventionally cast alloys																		
Rene 80	14	9.5	4	-	3	5	-	-	-	-	-	0.17	0.02	4	-	-	0.03	-
MAR-M200	9	10		-	5	2	-	-	-	-	-	0.15	0.015	12	1	-	0.05	-
MAR-M246	8.3	10	0.7	-	5.5	1	-	-	3	-	1.5	0.14	0.02	10	-	-	0.05	-
Inconel 713LC	12	-	4.5	-	5.9	0.6	-	-	-	-	-	0.05	0.01	-	2	-	0.1	-
Directionally cast alloys																		
Rene 80DS	12.9	9.6	4	-	3.02	4.48	-	-	-	-	0.074	0.07	0.015	6	-	-	0.02	-
CM247 LC DS	0.07	9.2	0.5	-	5.6	1	-	-	3	-	1.5	0.07	0.015	9.2	-	-	0.015	-
First generation single-crystal alloys																		
Rene N4	9.8	7.5	2	-	4.2	3.5	-	-	4.8	-	0.15	0.05	-	6	0.5	-	-	-
PWA 1480	10	5	-	-	5	1.5	-	-	12	-	-	-	-	4	-	-	-	-
CMSX-2	8	5	0.6	-	5.6	1	-	-	6	-	-	-	-	8	-	-	-	-
CMSX-3	8	5	0.6	-	5.6	1	-	-	6	-	0.1	-	-	8	-	-	-	-
RR2000	10	15	3	-	5.5	4	-	-	-	-	-	-	-	-	-	-	-	1 V
SRR99	8	5	-	-	5.5	2.2	-	-	3	-	-	-	-	10	-	-	-	-
Second generation single-crystal alloys																		
Rene N5	7	7.5	1.5	-	6.2	-	-	-	6.5	3	0.15	0.05	-	5	-	0.01	-	-
PWA 1484	5	10	2	-	5.6	-	-	-	9	3	0.1	-	-	6	-	-	-	-
CMSX-4	6.5	9	0.6	-	5.6	1	-	-	6.5	3	0.1	-	-	6	-	-	-	-
CMSX-6	9.8	5	3	-	4.8	4.7	-	-	2.1	-	-	-	-	-	-	-	-	-
SC180	5	10	2	-	5.2	1	-	-	9	3	0.1	-	-	5	-	-	-	-
Third generation single-crystal alloys																		
Rene N6	4.2	12.5	1.4	-	5.8	-	-	-	7.2	5.4	0.15	0.5	-	6	-	0.01	-	-
CMSX-10	2	3	0.4	-	5.7	0.2	-	-	8	6	0.2	-	-	5	0.1	-	-	-
TMS-75	3	12	2	-	6	-	-	-	6	5	0.1	-	-	6	-	-	-	-
TMS-113	2.9	11.9	2	-	6.6	-	-	-	6	6	0.1	-	-	6	-	-	-	-
TMS-121	3	6	3	-	6	-	-	-	6	5	0.1	-	-	6	-	-	-	-
Fourth generation single-crystal alloys																		
MC-NG	4	-	1	-	6	0.5	4	-	5	4	0.1	-	-	5	-	-	-	-
PWA 1497	2	16.5	2	-	5.6	-	3	-	8.3	6	0.15	-	-	6	-	-	-	-
TMS 138	3.2	5.8	2.8	-	5.9	-	2	-	5.6	5	0.1	-	-	5.9	-	-	-	-
TMS 138A	3.2	5.8	2.8	-	5.7	-	3.6	-	5.6	5.8	0.1	-	-	5.6	-	-	-	-
Fifth generation single-crystal alloys																		
TMS-162	3	5.8	3.9	-	5.8	-	6	-	5.6	4.9	0.1	-	-	5.8	-	-	-	-
TMS-173	3	5.6	2.8	-	5.6	-	5	-	5.6	6.9	0.1	-	-	5.6	-	-	-	-
TMS-196	4.6	5.6	2.4	-	5.6	-	5	-	5.6	6.4	0.1	-	-	5.6	-	-	-	-
Sixth generation single-crystal alloys																		
TMS-238	4.6	6.5	1.1	-	5.9	-	5	-	7.6	6.4	0.1	-	-	4	-	-	-	-
Wrought superalloys																		
Inconel 600	15.8	-	-	7.2	-	-	-	0.2	-	-	-	0.04	-	-	-	-	-	0.2 Si
Inconel 718	19	-	3	18.5	0.5	0.9	-	-	-	-	-	-	0.2	-	5.1	-	-	-
ATI-718 Plus	19	9	2.8	9	1.45	0.75	-	-	-	-	-	0.025	0.0055	1.1	5.6	-	-	-
Rene 41	19	11	10	-	1.5	3.1	-	-	-	-	-	0.09	0.005	-	-	-	-	-
Nimonic 80A	19.5	-	-	-	1.4	2.4	-	-	-	-	-	0.06	0.003	-	-	-	0.06	-
Nimonic 105	14.5	20	5	4.5	-	1.2	-	-	-	-	-	-	0.2	-	-	-	-	-
Waspaloy	19.5	13.5	-	-	1.3	3	-	-	-	-	-	0.08	0.006	4	-	-	0.03	-
Hastelloy X	22	1.5	9	6	18.5	-	-	0.5	-	-	-	0.1	-	6	-	-	-	0.5 Si
Hastelloy S	15.5	-	14.5	-	1	0.2	-	-	-	-	-	0.02	0.009	-	-	-	-	0.02 La
Udimet 500	18	18.5	4	-	2.9	3.9	-	-	-	-	-	0.08	0.006	-	-	-	0.05	-
Udimet 700	15	18.5	5.2	-	4.3	3.5	-	-	-	-	-	0.08	0.03	-	-	-	-	-
Powder-processed superalloys																		
Rene 95	13	8	-	-	3.5	2.5	-	-	-	-	-	0.065	0.013	3.5	3.5	-	0.05	-
Rene 88DT	16	13	-	-	2.1	3.7	-	-	-	-	-	0.03	0.015	4	0.7	-	-	-
Inconel 100	12.4	18.4	-	-	4.9	4.3	-	-	-	-	-	0.07	0.02	-	-	-	0.07	-
N18	11.2	15.6	-	-	4.4	4.4	-	-	-	-	0.5	0.02	0.015	-	-	-	0.03	-
Astroloy	14.9	17.2	5.1	-	4	3.5	-	-	-	0.04	-	0.025	-	-	-	-	0.04	-

## Data Availability

The data presented in this study are available on request from the corresponding authors.
